# Chemical mass shifts of cluster ions and adduct ions in quadrupolar ion traps revisited and extended

**DOI:** 10.1002/rcm.9436

**Published:** 2022-12-14

**Authors:** Joakim Romson, Åsa Emmer

**Affiliations:** ^1^ School of Engineering Sciences in Chemistry, Biotechnology and Health, Department of Chemistry, Division of Applied Physical Chemistry, Analytical Chemistry KTH Royal Institute of Technology Stockholm Sweden

## Abstract

**Rationale:**

Chemical mass shifts in quadrupolar ion traps have been studied previously but only for a limited number of analytes and mass ranges. Here, mass shifts of cluster ions, commonly used as calibrants, and other analytes are qualitatively evaluated on the Bruker amaZon spherical ion trap (QIT) and the Finnigan LXQ linear ion trap (LIT). To extend the mass range from previous experiments *m/z* up to 4000 are investigated.

**Methods:**

Chemical mass shifts of CsI, Y(HCOO)_3_, and NaCF_3_COO cluster ions, CF_3_COO^−^, Na^+^, and Cs^+^ adduct ions, protonated commercial calibration solutions and peptides, and deprotonated peptides were investigated on the Bruker amaZon speed QIT and some of these were also investigated on the Finnigan LXQ LIT.

**Results:**

On both instruments, peak distortions and mass shifts toward lower *m/z* became apparent as *m/z* approached 1000. To some extent, the issues were more severe at slower scans. Peak distortions included loss of resolution, tailing, or fronting and were different between the amaZon QIT and the LXQ LIT. The noncluster and nonadduct ions analyzed showed no obvious mass shifts or peak distortions under the same analysis conditions.

**Conclusions:**

As expected, the ion traps investigated here showed mass shift and peak distortion issues, and such issues persisted at *m/z* up to 4000 on both instruments. Peak distortions were different between the amaZon QIT and the LXQ LIT, and were not always visible despite mass shifts. Both mass shifts and peak distortions make cluster ions and some adduct ions unsuitable for ion trap calibration.

## 1 INTRODUCTION

Commercial ion traps commonly use nonideal, optimized, geometries to improve peak distortion, resolution, and minor mass shifts due to nondissociating collisions with buffer gas (damping) that may appear when using ideal geometries.^1^ The Finnigan LXQ uses a stretched geometry^2^ and the Bruker amaZon deviates from the ideal trap by using different asymptote angles.^3^ When comparing chemical mass shifts between different instruments, both trap geometries and applied electric potentials should be considered.

Chemical mass shifts of fragile ions due to ion dissociation during ejection from the trap in spherical ion traps (quadrupole ion traps, QITs) have long been known. These manifest as mass shifts toward lower *m/z*, peak broadening, and peak distortion, as shown on optimized traps.^4^, ^5^, ^6^ The shifts have been shown to depend on, for example, buffer gas pressure in both optimized^5^, ^6^, ^7^ and ideal^7^ traps, the effect being less severe in the optimized case.^7^ The same molecule may show different stability depending on its ionization, that is, deprotonated or adducted.^4^, ^6^, ^8^ In addition to severe loss of resolution, some fronting could be seen for a peptide when using Ar instead of He as buffer gas in a QIT. Being heavier, Ar showed higher collision‐induced dissociation (CID) efficiency than He in an optimized trap.^9^ This suggests the possibility that using Ar instead of He gives (more) unintentional dissociation during ejection. Linear ion traps (LITs) operate on the same principles as QITs but have been less investigated.

Analyses in ion traps are commonly done by scanning from lower to higher *m/z* (forward scan), but analysis can also be performed scanning from higher to lower *m/z* (reverse scan). It appears that reverse scans require lower auxiliary field amplitudes compared to forward scans for optimal ejection, thus reverse scanning may in some cases be used for ejection with less dissociation and less mass shift of fragile ions. However, some loss of resolution has been observed for the reverse scans.^6^, ^10^ Additionally, the shift may be toward higher *m/z* in the reverse scan.^10^

In other cases not discussed here, (minor) chemical mass shifts have been attributed to nondissociating collisions with buffer gas (damping) and depend on collisional cross‐section. Such shifts show opposite signs for forward and reverse scans, respectively, so it has been proposed that accuracy can be increased by combining forward and reverse scans.^11^

Ion traps commonly operate in the lower *m/z* ranges (*m/z* < 3000), but extended mass range modes in commercial traps may allow analysis to *m/z* 6000, but little experimental investigation has been performed on mass shifts at high *m/z*. For calibration of high *m/z* ranges in elecrospray ionization mass spectrometry (ESI‐MS), cluster ions are the most common calibrants.^12^ Despite the evidence of mass shifts found here, Bruker suggests using dimers or clusters for high *m/z* calibration of the amaZon speed QIT.^13^ The manual of the Finnigan LXQ LIT shows the use of high molecular weight poly(propyleneglycol) for calibration,^14^ but in preliminary studies on similar molecules we failed to obtain high *m/z* signals and experienced memory effects, so that was not tested here. Instead, high mass dendrimers found to yield high *m/z* signals were used for comparison to cluster ions at *m/z* 2000–4000. Despite the potentially severe memory effects of poly(propyleneglycol) and poly(ethyleneglycol),^12^ they may be more suitable for high *m/z* calibration of ion traps than the dimers suggested by Bruker.

## 2 EXPERIMENTAL

### 2.1 Chemicals

Peptides, tetrahydrofuran (THF), dimethyl sulfoxide (DMSO), ESI tuning mix (perfluorinated phosphazenes), and CsI were from Sigma Aldrich. MeOH and NaHCOO were from Merck, MeCN was from Fisher, and HCOOH was from Fluka. Pierce™ LTQ ESI Positive Ion Calibration Solution (“Pierce”, based on perfluorinated phosphazenes, with caffeine and peptide Met‐Arg‐Phe‐Ala [MRFA]) was from Thermo Scientific. Water was purified in a Synergy 185 system (Millipore) to a resistivity of 18.2 MΩ.cm. Dendrimers were provided by Polymer Factory Sweden AB.

### 2.2 Instrumentation

The QIT used was the Bruker amaZon speed RF 0212G003 2013 spherical ion trap. Its ring electrode main radio frequency (RF) is 781 kHz, with the endcap auxiliary RF 1/3 of this. It was operated using TrapControl 8.0.25 (Bruker Daltonik).

The LIT was the Finnigan LXQ 10149 linear ion trap. In this trap, the X‐rods (with ejection slits) are distanced 4.76 mm from the trap center, while the Y‐rods are 4 mm from the center. Its main RF is 1.2 MHz. It was operated using Tune Plus 2.2 (Thermo Electron).

Both instruments used He as buffer gas. The pressure readings are for the high vacuum chamber, not the trap itself, where the pressure is higher.

### 2.3 Sample preparation

CsI was dissolved in MeCN to 5 mM (adapted from^15^). Yttrium formate (Y(HCOO)_3_) was prepared by suspending 200 mg/ml Y_2_O_3_ in HCOOH, then diluting 100× into 9:1 MeOH:H_2_O.^8^ ESI tuning mix stock solution was diluted 20× in 19:1 MeCN:H_2_O (5× recommended concentration). Pierce was used as delivered or, to obtain CsI‐clusters simultaneously as the calibrant signals, mixed with 5 mM CsI in MeCN 9:1 (amaZon) or 4:1 (LXQ). The peptide mixture consisted of Leucine‐enkephalin, Angiotensin II, Angiotensin I, Bombesin, and adrenocorticotropic hormone (ACTH) amino acids18–39 at 0.8 μM each, in 3:2 MeCN:H_2_O, with 0.1 vol% HCOOH. Low mass Na^+^‐adducted dendrimers (SpheriCal®‐ESI) were prepared according to the literature.^16^ The high‐mass dendrimer mixture consisted of four dendrimers, C_128_H_240_O_18_ (“D2089”), C_52_H_34_O_13_I_12_ (“D2412”), C_100_H_82_O_30_I_10_ (“D3055”), and C_124_H_106_O_37_I_12_ (“D3732”), 100 μg/ml each, with 5 μg/ml NaHCOO, dissolved in THF:DMSO 1:1 and injected as such. Cs^+^ adducts of the dendrimers were obtained by adding 2 vol% 5 mM CsI. On the LXQ, both Na^+^, and Cs^+^ adducts were obtained simultaneously by infusion of 5 mM CsI in MeCN without rinsing before infusion of the dendrimer mixture.

### 2.4 Analysis

To a large extent, scans were performed as during regular use, meaning mostly standard/default parameters and forward scan without isolation of ions.

All spectra were re‐calibrated against a seemingly relatively stable ion series after acquisition (quadratic functions were used for all re‐calibrations) and shifts shown are thus those relative to the calibrants. Calibration results such as curve fit and residual error can be seen in supporting information ‐ supplementary calibration fit. The LXQ low mass range was calibrated against Pierce (default calibration solution, *m/z* 195, 524, 1,222, 1,522, 1822). Positive mode spectra on the amaZon were calibrated against ESI tuning mix (default calibration solution, *m/z* 118, 322, 622, 922, 1,522, 2,122, 2,722) and negative mode spectra against a peptide mixture (*m/z* 554, 809, 1,045, 1,232, 1,295, 1,618). The high *m/z* ranges of both the LXQ and the amaZon were calibrated against the four Na^+^‐adducted dendrimers (*m/z* 2089, 2,412, 3,055, 3,732). Since it is not certain that the Na^+^‐adducted dendrimers show no shift themselves, it must be emphasized that all shifts shown are relative. Reported *m/z* values are averages of three spectra (if not detected in three spectra, the datapoint was skipped) and error bars show one standard deviation. All peak shapes shown are from averaging several spectra.

On the amaZon, ion charge control (ICC) was set to 100 000 (200 000 default) and 20 scans/spectrum, and on the LXQ automatic gain control (AGC) was set to 15 000 and 10 scans/spectrum. On the amaZon, Xtreme (52 000 u/s), Ultra (32 500 u/s), Enhanced resolution (8100 u/s), and Maximum resolution (5200 u/s) scan modes were used for *m/z* 100–3000, and for *m/z* 2000–4000 Extended mass range (27 000 u/s) scan mode (the only available mode for the high mass range) was used. The default values used for low mass cutoffs were *m/z* 100, 70, 50, 50, and 200 for Xtreme, Ultra, Enhanced resolution, Maximum resolution, and Extended mass range, respectively. He pressure difference to setpoint was 1.80 × 10^−9^ bar for all modes except Maximum resolution, were it was 0.90 × 10^−9^ bar.

On the LXQ, Turbo (128 000 u/s), Normal (16 600 u/s), Enhanced (5000 u/s), and Zoom (1100 u/s) scan modes were used for *m/z* 150–2000, and for *m/z* 2000–4000 Turbo, Normal, and Zoom scan modes were used (Enhanced scan mode is not available for the high mass range). The default values used for low mass cutoffs were *m/z* 50 for all scan speeds in the normal mass range and 100 for all scan speeds in extended mass range. The high vacuum was 9.7 × 10^−9^ bar.

For the low mass range, Y(HCOO)_3_, NaCF_3_COO‐clusters, protonated and CF_3_COO^−^‐adducted ESI tuning mix, Na^+^‐adducted low mass dendrimers of SpheriCal®‐ESI, and protonated and deprotonated peptides were analyzed in all four scan speeds on the amaZon. CsI‐clusters and the CsI + Pierce mixture were analyzed in all four scan speeds of both the amaZon and the LXQ, and protonated Pierce on the LXQ.

In the Extended mass range mode on the amaZon, the four dendrimers as Na^+^ and Cs^+^ adducts, and CsI clusters were analyzed. In high mass range on the LXQ, the four dendrimers as both Na^+^ and Cs^+^ adducts in the same spectra, and CsI clusters were analyzed in all three scan speeds. The experiments are summarized in Table 1.

**TABLE 1:** Summary of experiments

	Range	*m/z*	Calibrants	Analytes	Speed	u/s
**LXQ LIT**	Low	150–2000	Pierce + H^+^	CsI (CsI + Pierce)	Turbo	128 000
					Normal	16 000
					Enhanced	5000
					Zoom	1100
	High	2000–4000	High mass dendrimers + Na^+^	CsI (high‐mass dendrimers + Na^+^ + Cs^+^)	Turbo	128 000
					Normal	16 000
					Zoom	1100
**amaZon QIT**	Low	100–3000	Positive mode: ESI tuning mix + H^+^ Negative mode: peptides − H^+^	Positive mode: ESI tuning mix + H^+^ NaCF_3_COO CsI (CsI + Pierce) SpheriCal®‐ESI + Na^+^ Peptides + H^+^ Negative mode: Peptides − H^+^ ESI tuning mix + CF_3_COO^−^ Y(HCOO)_3_	Xtreme	52 000
					Ultra	32 500
					Enhanced resolution	8100
					Maximum resolution	5200
	High	2000–4000	High mass dendrimers + Na^+^	CsI High mass dendrimers + Na^+^ High mass dendrimers + Cs^+^	Extended mass range	27 000

Theoretical *m/z* values were calculated from recently published masses and weights.^17^, ^18^, ^19^ Apex peak picking (default) was used for isotope resolving scans on the amaZon, otherwise the centroid was used (default on the LXQ). This means that Apex was used also when isotope resolution was lost on some fragile ions during Xtreme, Ultra, Enhanced, and Maximum resolution scans on the amaZon, which means that the mass shifts for nonmonoisotopic ions can only be qualitatively interpreted. For the monoisotopic CsI clusters, isotope resolution is irrelevant. In DataAnalysis, the Apex algorithm does not choose the single highest point but interpolates a few points around it to make a fitted maximum, allowing for noisy peaks. On the LXQ, isotope resolution was never lost for fragile ions so the mass shifts are more certain. A few peaks showing severe splitting (such that two peaks were detected in a single one) on either instrument were slightly smoothed to facilitate peak picking (no smoothing has been applied to any shown peak shapes). For peaks with a Gaussian shape the apex and centroid give the same value, but with more asymmetry the difference becomes larger. With significant asymmetry, apex peak picking might be more accurate.^4^ As can be seen in Figures 4 and 5, using apex instead of centroid on the LXQ may not necessarily eliminate mass shifts in this study.

Calculated average mass was used in Turbo scan and Extended mass range scan, and for multiply charged peptides when isotope clusters were not resolved. In all other cases, the calculated monoisotopic mass was used. amaZon spectra were processed in DataAnalysis 4.2 (Bruker Daltonik) and LXQ spectra were processed in Qual Browser 2.0 (Thermo Electron). Data analysis was performed in LibreOffice Calc 6 and 7.

## 3 RESULTS AND DISCUSSION

Although some signals were of low intensity and some spectra were noisy, we refrained from measuring these again to avoid bias; it could be argued that a mix of high intensity and low intensity signals is closer to real applications. However, all data from 1 day was discarded as instrument performance in terms of resolution and sensitivity was unsatisfactory, probably due to unstable He pressure after start‐up. Importantly, all mass shifts discussed are relative the calibration ions. In the absence of mass shifts, it is expected that analytes at similar *m/z* show similar absolute *m/z* errors. In addition to chemical mass shift toward lower *m/z* and peak distortion for fragile ions, some mass shift toward higher *m/z*, loss of resolution, and tailing can be expected for high‐intensity signals.^20^, ^21^ To limit this, automatic gain control was used on both instruments, set to values that did not show significant space charge effects during normal use (as can be seen on the peak shapes of stable ions). It may be noted that larger *m/z* ions experience less space charge as the lower *m/z* ions are ejected before them.

**FIGURE 1:**
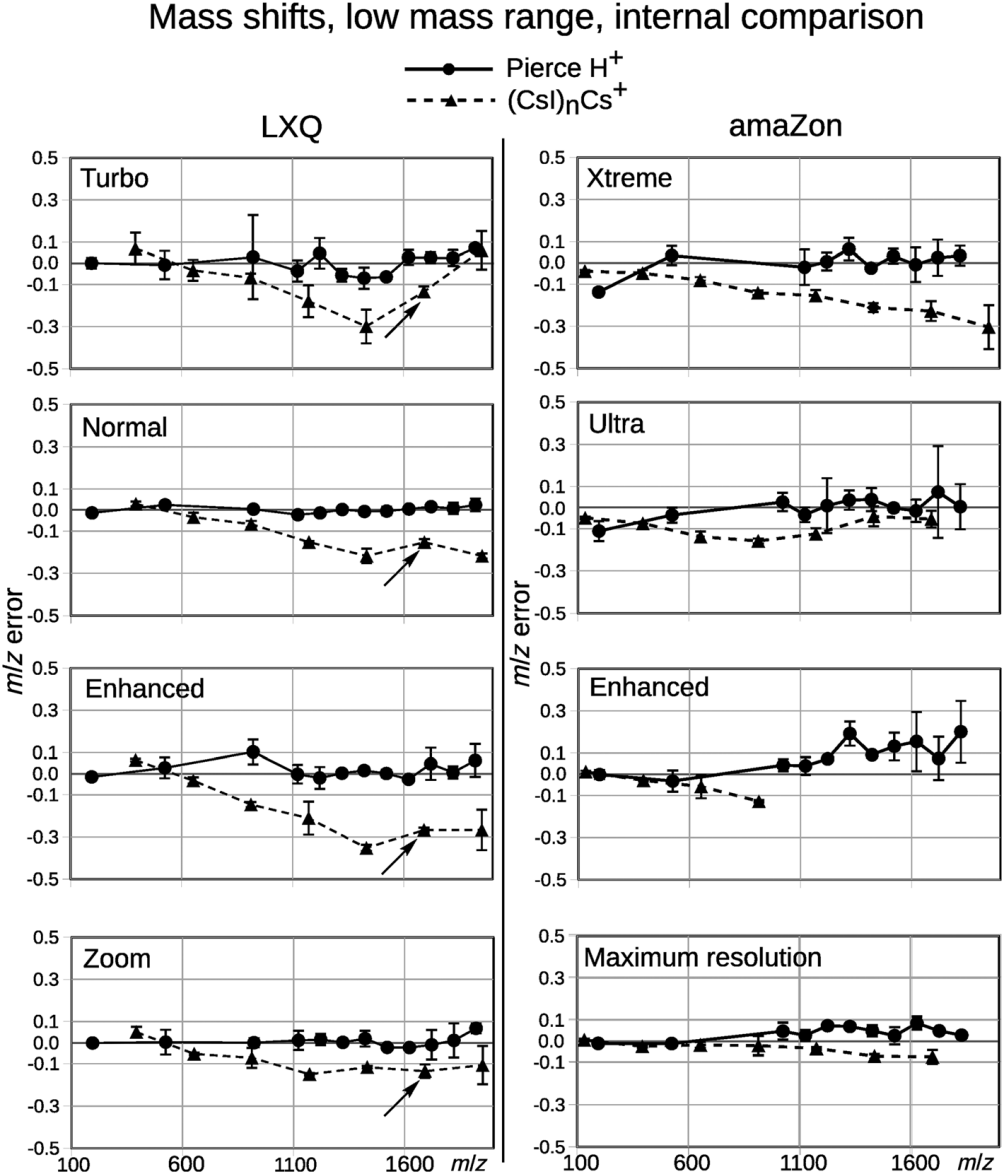
*m/z* errors of CsI clusters and Pierce LTQ positive calibration solution, both in the same spectra, at four different scan speeds, from fast (top) to slow (bottom), on both the LXQ LIT and the amaZon QIT. Arrows mark (CsI)6Cs^+^, a more stable cluster (see text), in the LXQ spectra. The severe distortion of CsI cluster peaks in the amaZon (see Figure 3) and accompanying uncertainties in peak picking explain the sometimes erratic measurements and mean that the magnitude of the shifts may vary. amaZon mass shifts should be interpreted with caution.

**FIGURE 2:**
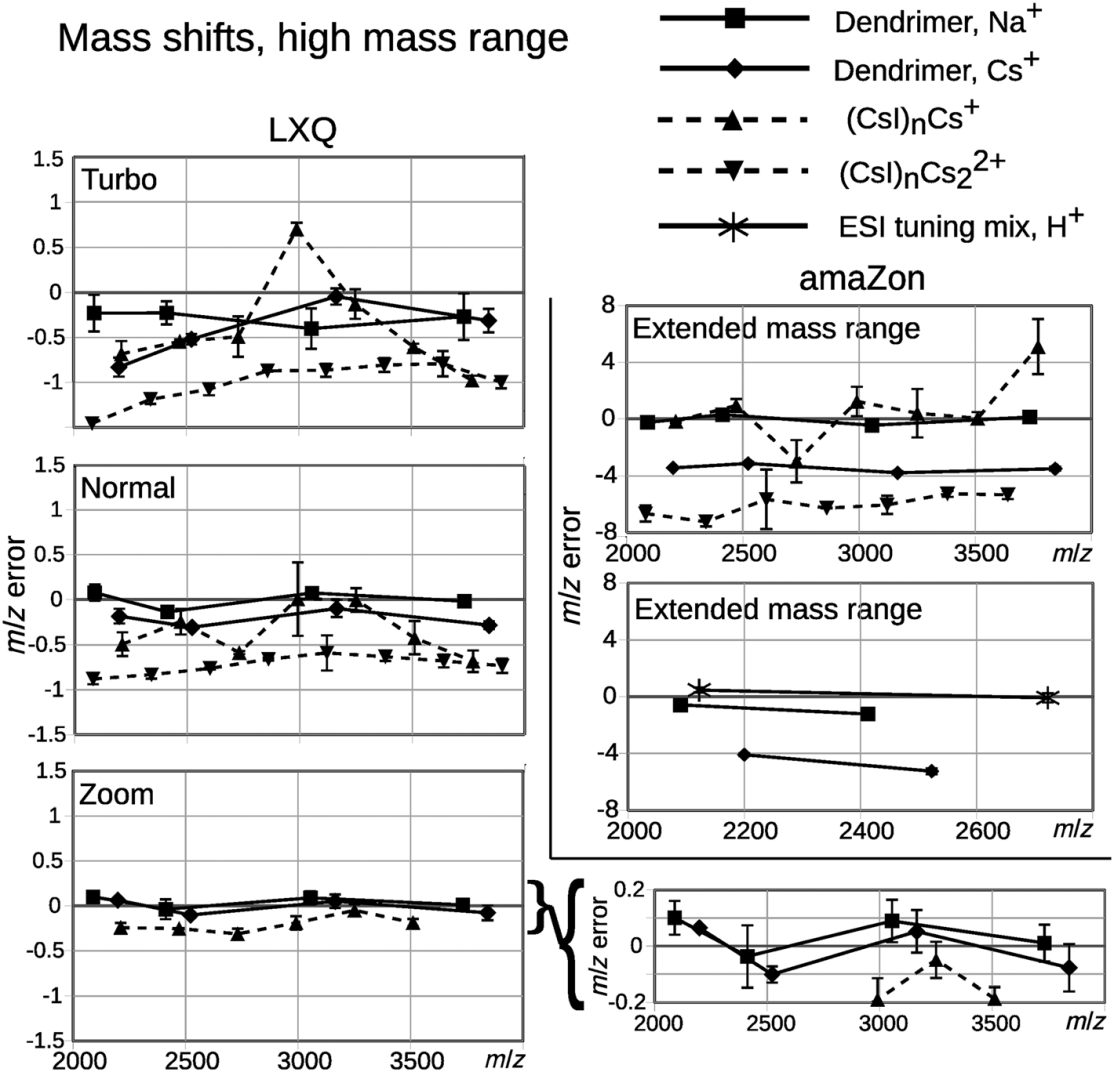
*m/z‐*errors in the high mass range on the LXQ LIT, three scan speeds, and the amaZon QIT, one scan speed. On both instruments, *m/z* errors of the dendrimers as Na^+^ adducts and Cs^+^ adducts, and CsI clusters are compared. Additionally, on the amaZon, two dendrimers as Na^+^ and Cs^+^ adducts are compared to the two highest signals of ESI tuning mix.

**FIGURE 3:**
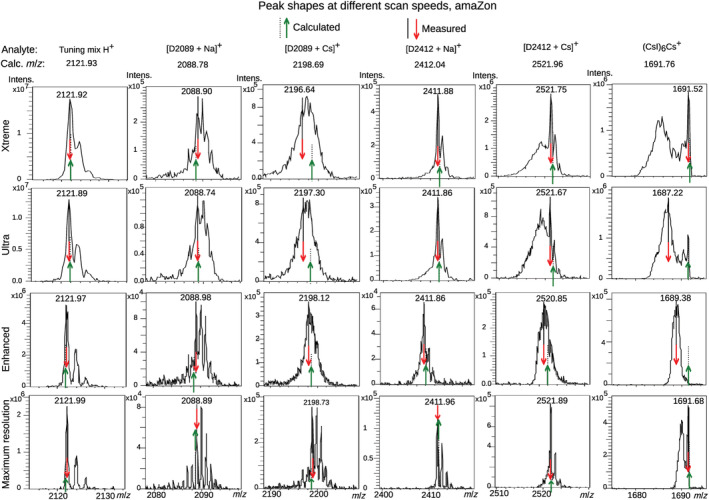
Peak shapes of protonated ESI‐tuning mix and Na^+^‐adducted dendrimers, Cs^+^‐adducted dendrimers, and a CsI cluster at different scan speeds on the amaZon QIT. Calculated and measured *m/z* are shown with dashed and solid lines, and with green/up arrows and red/down arrows, respectively. Each spectrum is the average of several scans. No smoothing or baseline subtraction has been applied. [Color figure can be viewed at wileyonlinelibrary.com]

**FIGURE 4:**
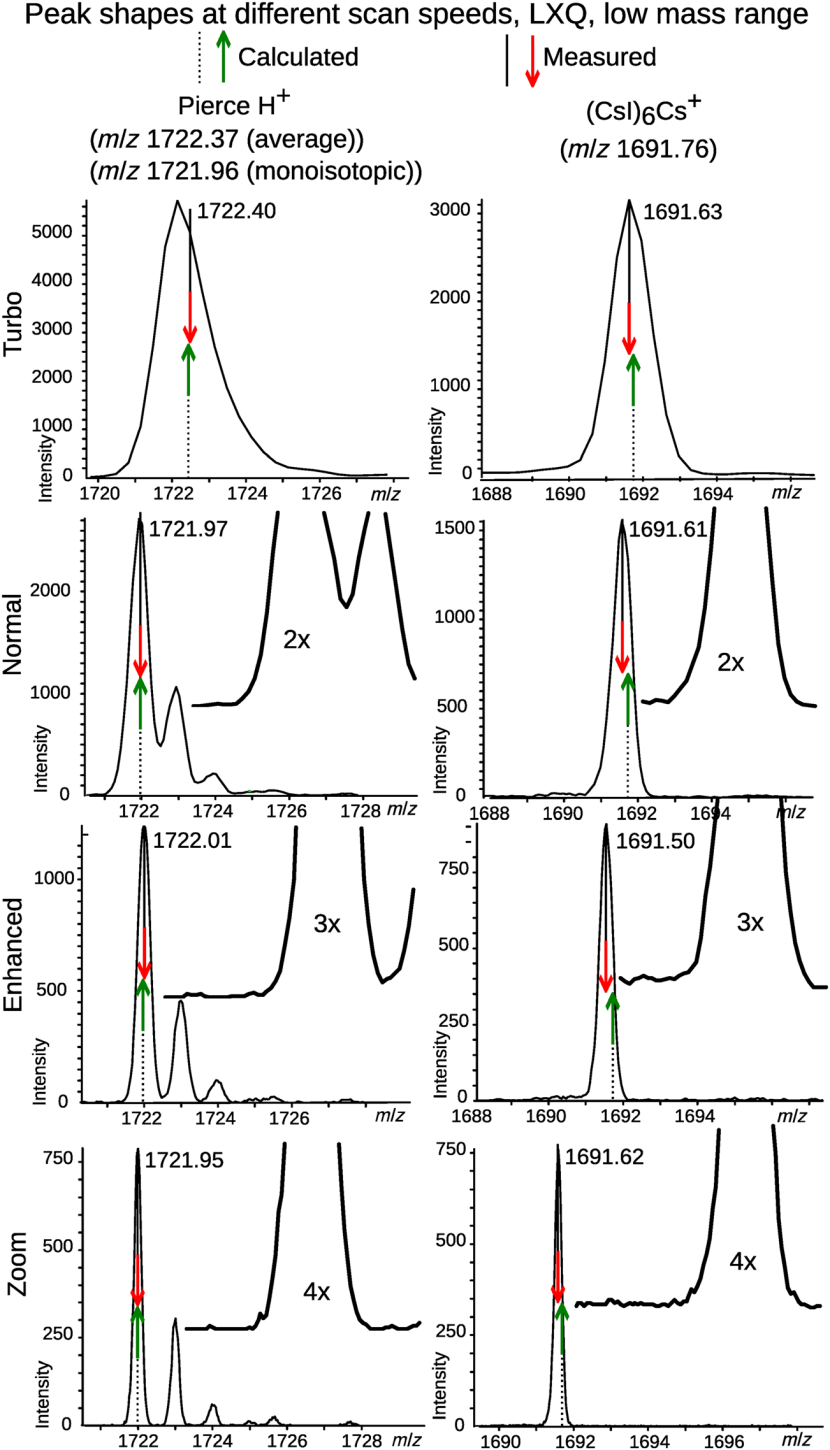
Comparison of peak shapes for protonated Pierce LTQ positive ion calibration solution and a singly charged CsI cluster in the low mass range on the LXQ LIT at four different scan speeds. Insets in rows 2, 3, and 4 show closeups of the peak shapes at 2×, 3×, and 4× magnification, respectively. Calculated and measured *m/z* are shown with dashed and solid lines, and with green/up arrows and red/down arrows, respectively. Each spectrum is the average of several scans. No smoothing or baseline subtraction has been applied. [Color figure can be viewed at wileyonlinelibrary.com]

**FIGURE 5:**
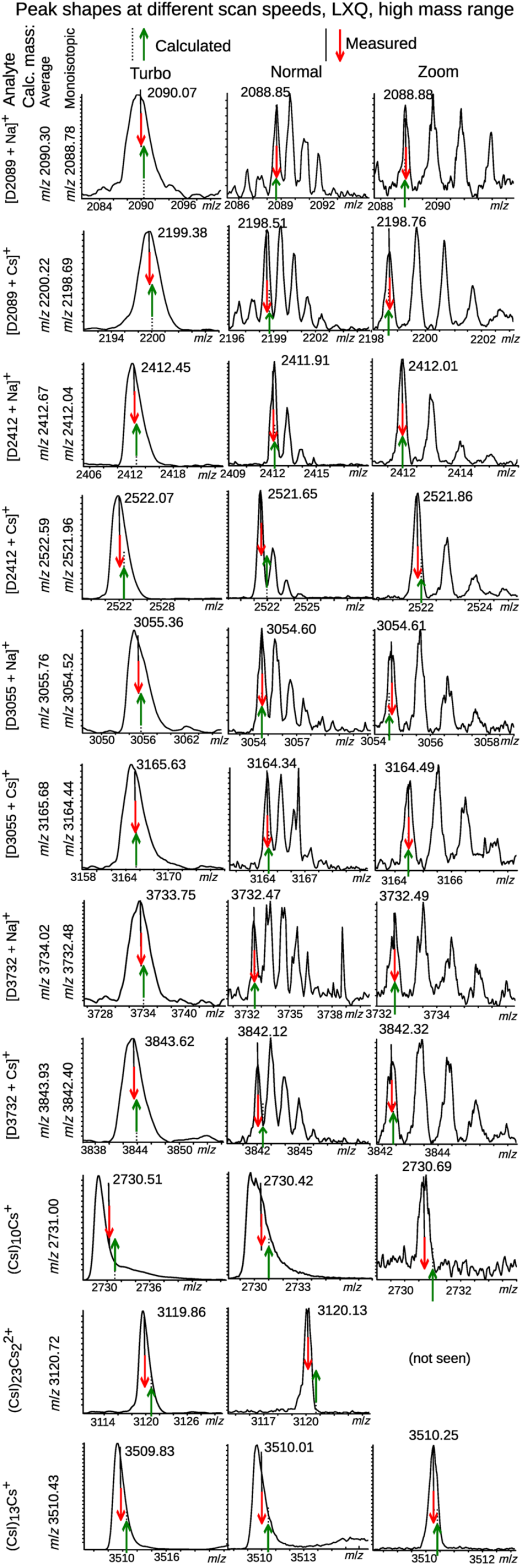
Comparison of peak shapes in the high mass range on the LXQ LIT for two singly charged and one doubly charged CsI‐cluster, and Na^+^‐ and Cs^+^‐adducted dendrimers, at three different scan speeds (the doubly charged CsI cluster could not be seen in Zoom scan mode). Note the different *m/z* scales, where Normal and Zoom scan spectra show 2× and 4× closeup compared to Turbo scan spectra, respectively. Calculated and measured *m/z* are shown with dashed and solid lines, and with green/up arrows and red/down arrows, respectively. Each spectrum is the average of several scans. No smoothing or baseline subtraction has been applied. [Color figure can be viewed at wileyonlinelibrary.com]

For the amaZon, the mass shifts and peak distortions of the CsI clusters in positive mode were representative of the other cluster ions and some of the adducts (as can be seen in supporting information ‐ Supplementary mass shifts, Figures 5–12). For the LXQ, the mass shifts from the internal comparison showed similar behavior to the mass shifts from the external mass comparisons, although they were much less pronounced in the external comparison (see supporting information ‐ Supplementary mass shifts, Figures 1–4). The difference may be due to differences in peak intensities, with CsI clusters showing very intense signals. Negative mode, evaluated on the amaZon, showed similar results to positive mode (see supporting information ‐ Supplementary mass shifts, Figures 13–20), therefore mainly results from CsI clusters and positive mode are presented here.

### 3.1 Mass shifts

From Figures 1 and 2, and in supporting information ‐ Supplementary mass shifts, Figures 1–20, it can be seen that analytes that showed chemical mass shifts generally had shifts toward lower *m/z* (relative to the calibration solution used) in the higher *m/z* ranges in at least some scan modes. All cluster ions analyzed, and some of the adduct ions, showed mass shifts in the higher *m/z* ranges. Mass shifts at lower *m/z* ranges (*m/z* < 1000), if present, were less clear. Adduct ions that showed shifts included CF_3_COO^−^‐adducted ESI tuning mix signals (see supporting information ‐ Supplementary mass shifts, Figures 13–20).

The CsI cluster at *m/z* 1692 (CsI)_6_Cs^+^ has previously been shown to be more stable in CID compared to other clusters in the range,^22^ and looking at the data from the LXQ (Figure 1, left, marked with arrows) (amaZon cluster peaks were severely distorted; Figure 3), this *m/z* shows less shift compared to the nearby analytes. Interestingly, there were large differences between the mass shifts of (CsI)_n_Cs^+^ and (CsI)_n_Cs_2_^2+^ on both instruments, with the latter showing larger shift toward lower *m/z* (Figure 2). Doubly charged CsI clusters were almost exclusively seen in the high mass range modes.

Roughly, shifts were larger at slower scan speeds for both instruments, but the effect appeared smaller than in preliminary studies.^12^ The Maximum resolution mode on the amaZon and the Zoom‐scan mode on the LXQ did not follow this trend, and the mass shifts were instead smaller.

Protonated, deprotonated, and Na^+^‐adducted analytes showed no obvious mass shifts relative to the calibration solution used (see supporting information ‐ Supplementary mass shifts Figures 5–20), except in the extended mass range where two of the high‐mass dendrimers showed error toward lower *m/z* relative to protonated ESI tuning mix (Figure 2). In line with these results, previous studies on lower mass Na^+^‐adducted dendrimers of SpheriCal®‐ESI showed no mass shifts relative to peptides, and no peak distortion, on the amaZon.^16^ The Cs^+^‐adducted high‐mass dendrimers showed mass shifts compared to the Na^+^‐adducted dendrimers, but not as severe as the shifts of some of the cluster ions (Figure 2). As it has been shown that, for example, protonated and Na^+^‐adducted analytes had different stabilities,^4^, ^6^, ^8^ we assume that the difference found here between Na^+^ adducts and Cs^+^ adducts has a similar origin.

### 3.2 Peak shapes

Although the mass shifts were similar on both instruments, peak distortions were different. In the low mass range on the amaZon, cluster ions and some adduct ions showed increasing fronting, which became more severe for larger clusters and slower scans, again except for the Maximum resolution scan. Eventually, the “true” cluster peak was overshadowed by a very broad noisy peak at lower *m/z* (see Figure 3, rightmost, and supporting information ‐ Supplementary peak shapes, Figures 1–5, 11–15, 21–25, and 32–36). Notably, this includes the CF_3_COO^−^‐adducted ESI tuning mix peaks (see supporting information ‐ Supplementary peak shapes, Figures 3, 13, 23, and 34) and CF_3_COO^−^‐adducted Pierce negative ion calibration solution.^12^ This severe peak distortion obviously complicates peak picking, and thus the mass shifts in the amaZon should not be quantitatively interpreted. Indeed, the distortion renders cluster ions unsuitable for calibration at higher *m/z*, disregarding the mass shift. As expected, other analytes showed improved resolution with decreased scan speed (see supporting information ‐ Supplementary peak shapes, Figures 6, 16, 26–27, 37–38), and although the resolution of Na^+^‐adducted D2089 and D2412 improved, they showed lower resolution than expected (Figure 3, columns 2 and 4), possibly indicating lability. On the amaZon, *m/z* above 3000, that is, the two heavier dendrimers, could only be analyzed with one scan speed and without isotope resolution.

In the Extended mass range on the amaZon, (CsI)_n_Cs^+^ clusters showed severe tailing and very broad peaks (see supporting information ‐ Supplementary peak shapes, Figure 51), while peak widths of (CsI)_n_Cs_2_^2+^ clusters (see supporting information ‐ Supplementary peak shapes, Figure 52) were similar to those of the dendrimers (see supporting information ‐ Supplementary peak shapes, Figures 43–50). Although Na^+^‐adducted D3732 showed fronting at the base of the peak there was no additional obvious difference in peak shape between Na^+^‐ and Cs^+^‐adducted dendrimers, despite the Cs^+^ adducts showing shifts to lower *m/z* compared to the Na^+^ adducts.

In the low mass range on the LXQ, CsI cluster ions showed fronting, increasing with decreasing scan speed, again except for the Zoom scan (Figure 4, right), while no peak distortion was seen in protonated Pierce peaks (Figure 4, left). However, peak distortion was much less severe on the LXQ than on the amaZon. Despite the mass shifts, peak widths of CsI clusters decreased with slower scans (Figure 4, right), which is different from the amaZon, where this trend was less clear (Figure 3, rightmost and in supporting information ‐ Supplementary peak shapes, Figures 4, 14, 24, and 35).

In the high mass range on the LXQ there was no obvious difference in peak shape between Na^+^‐ and Cs^+^‐adducted dendrimers (see supporting information ‐ Supplementary peak shapes, Figure 53), despite the mass shifts. It was surprising to see that (CsI)_n_Cs^+^ cluster peaks showed fronting in the low mass range (Figure 4, right), but tailing in the high mass range (Figure 5, rows 9 and 11). However, (CsI)_n_Cs_2_^2+^, seen in the high mass range in Turbo and Normal scans (not seen in the Zoom scan), showed tailing in Turbo scan, but fronting in Normal scan (Figure 5, row 10). The tailing of the (CsI)_n_Cs^+^ cluster signals in the high mass range shifted the centroid toward higher *m/z* relative to the peak apex, and this lowered the measured mass shift.

One possible explanation for the much more severe peak distortion on the amaZon QIT could be the “pre‐excitation” used to increase trap capacity.^23^ Although the Maximum resolution mode on the amaZon operates at reduced He pressure, it appears that the Zoom scan on the LXQ does not as no change in the high‐vacuum gauge readout was seen, and thus the reduced distortion and reduced mass shifts in these scan modes cannot be fully explained by He pressure. However, it could be speculated that these high‐resolution scan modes operate at a lower amplitude of the auxiliary frequency compared to lower resolution scan modes to optimize resolution and sensitivity, respectively.^24^, ^25^

## 4 CONCLUSIONS

The results of the qualitative study indicate that shifts were generally present at higher *m/z* and, except for the slowest scans on both instruments, were to some extent worse with decreasing scan speed. In total, these results, except for the slowest scans, correspond well to the previous studies on mass shifts in QIT.

Some peak distortion often accompanied mass shifts, but was different between the amaZon QIT and the LXQ LIT. Importantly, mass shifts did not always correlate well with peak distortion. For example, on the LXQ the Cs^+^‐adducted dendrimers showed a negative mass shift relative the Na^+^ adducts without obvious difference in peak shape or resolution. In the Extended mass range scan on the amaZon (CsI)_n_Cs_2_^2+^ cluster signals showed narrow peak shapes, but the largest mass shifts, while (CsI)_n_Cs^+^ cluster signals showed severe tailing and broadening, yet smaller mass shift. Additionally, while peak distortion was more severe on the amaZon QIT, mass shifts were not always worse. Thus, it appears that it is not always possible to predict mass shift severity from the peak shape.

This suggests that ion trap users should look for peak distortions and mass shifts especially during high *m/z* analysis and high‐resolution measurements. The latter may be of additional importance since high resolution is often associated with high accuracy, while in some instances it may instead result in worse accuracy for fragile ions. Notable, however, is the relatively good performance of the Maximum resolution and the Zoom scans compared to the Enhanced scans.

All analyzed heavy cluster ions and some adduct ions showed at least some mass shifts. Cluster ions seem unsuitable for calibration of ion traps and adducts should only be used with caution. Similarly, analysis of such ions may yield inaccurate results. For suitable calibrants, we refer to a recent review (Mass calibration options for accurate ESI‐MS).^12^

## CONFLICTS OF INTEREST

The authors declare no conflicts of interest.

## ASSOCIATED CONTENT

Three documents with supporting information are included: Supplementary calibration fit contains more detailed results and evaluation of the calibrations. Supplementary mass shifts contains plots of mass shifts not shown in the main article. Supplementary peak shapes shows more peak shapes in addition to those shown in the main article.

### PEER REVIEW

The peer review history for this article is available at https://publons.com/publon/10.1002/rcm.9436.

## Supporting information


**DATA S1** Supplementary calibration fit

**DATA S2** Supplementary mass shifts

**DATA S3** Supplementary peak shapes

## Data Availability

The data that support the findings of this study are available from the corresponding author upon reasonable request.
