# Supplementary material for: Chemical mass shifts of cluster ions and adduct ions in quadrupolar ion traps revisited and extended
Source: Rapid Commun Mass Spectrom. 2022 Dec 14;37(3):e9436. doi: 10.1002/rcm.9436 (PMC10078176; doi:10.1002/rcm.9436)
Supplement: Supplementary file 1 — DATA S1 Supplementary calibration fit [file RCM-37-0-s003.pdf]

Supplementary - Calibration fit

Table 1: Residual errors for the different calibrations of the LXQ and the amaZon

Instrument	Calibration	Scan	Residual error standard deviation [m/z]
LXQ	Pierce	Turbo	0.038
		Normal	0.015
		Enhanced	0.017
		Zoom	0.013
	High mass dendrimers	Turbo	0.112
		Normal	0.080
		Zoom	0.087
amaZon	ESI tuning mix	Xtreme	0.021
		Ultra	0.048
		Enhanced	0.047
		Maximum resolution	0.029
	Peptides (negative mode)	Xtreme	0.104
		Ultra	0.079
		Enhanced	0.043
		Maximum resolution	0.045
	High mass dendrimers	Extended mass range	0.297

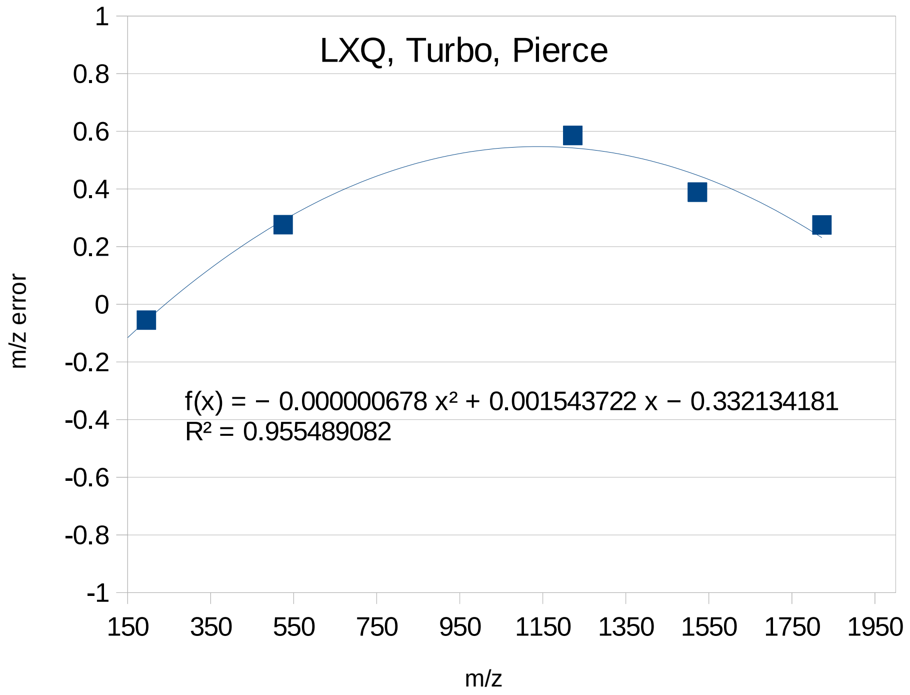
LXQ, Turbo, Pierce
f(x) = − 0.000000678 x² + 0.001543722 x − 0.332134181
R² = 0.955489082
m/z error
m/z
1
0.8
0.6
0.4
0.2
0
-0.2
-0.4
-0.6
-0.8
-1
150
350
550
750
950
1150
1350
1550
1750
1950

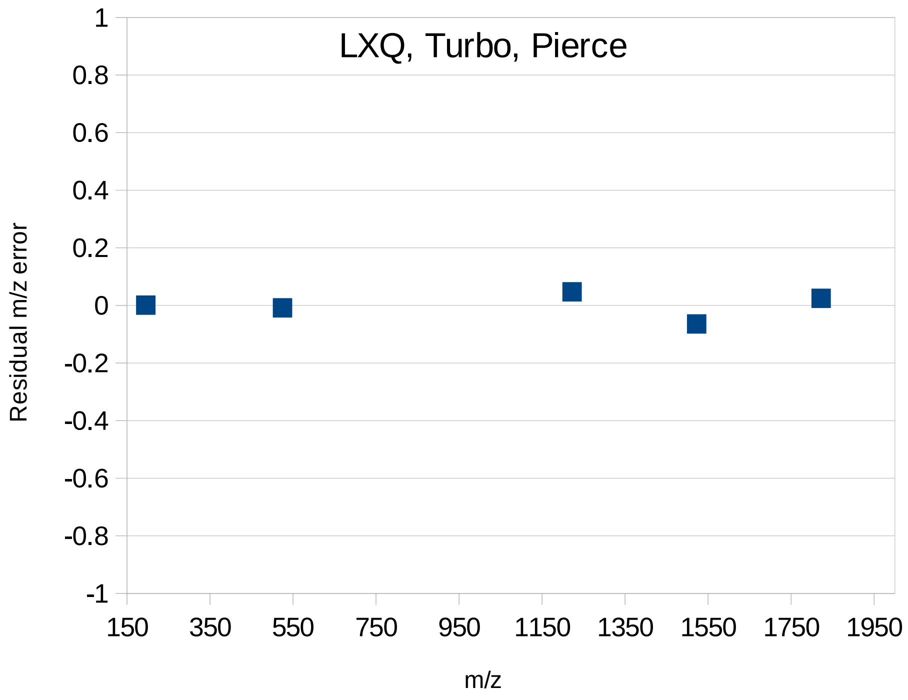
LXQ, Turbo, Pierce
Residual m/z error
m/z
1
0.8
0.6
0.4
0.2
0
-0.2
-0.4
-0.6
-0.8
-1
150
350
550
750
950
1150
1350
1550
1750
1950

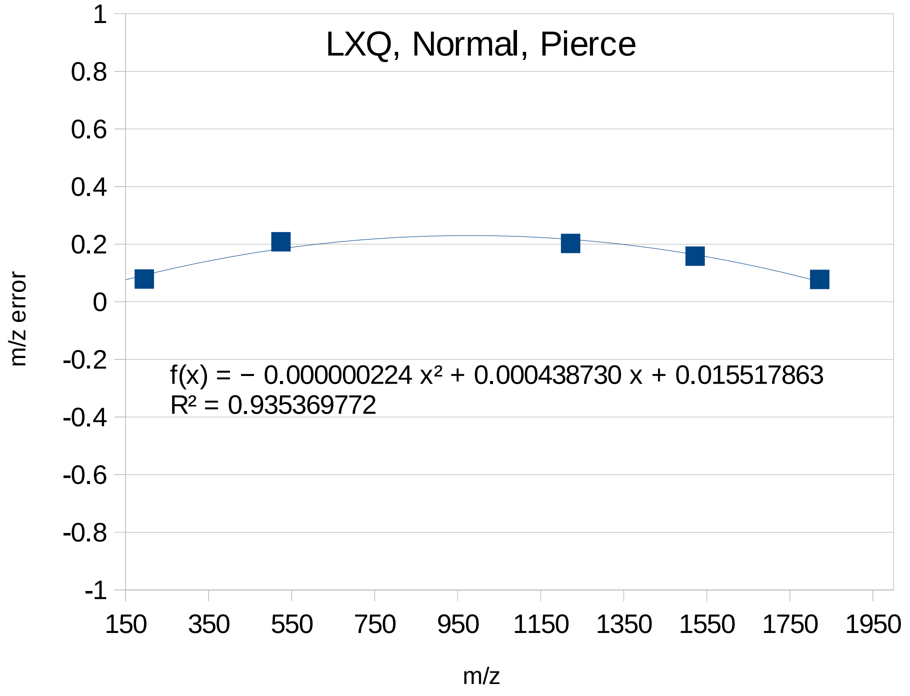
LXQ, Normal, Pierce
f(x) = − 0.000000224 x² + 0.000438730 x + 0.015517863
R² = 0.935369772
m/z error
m/z
1
0.8
0.6
0.4
0.2
0
-0.2
-0.4
-0.6
-0.8
-1
150 350 550 750 950 1150 1350 1550 1750 1950

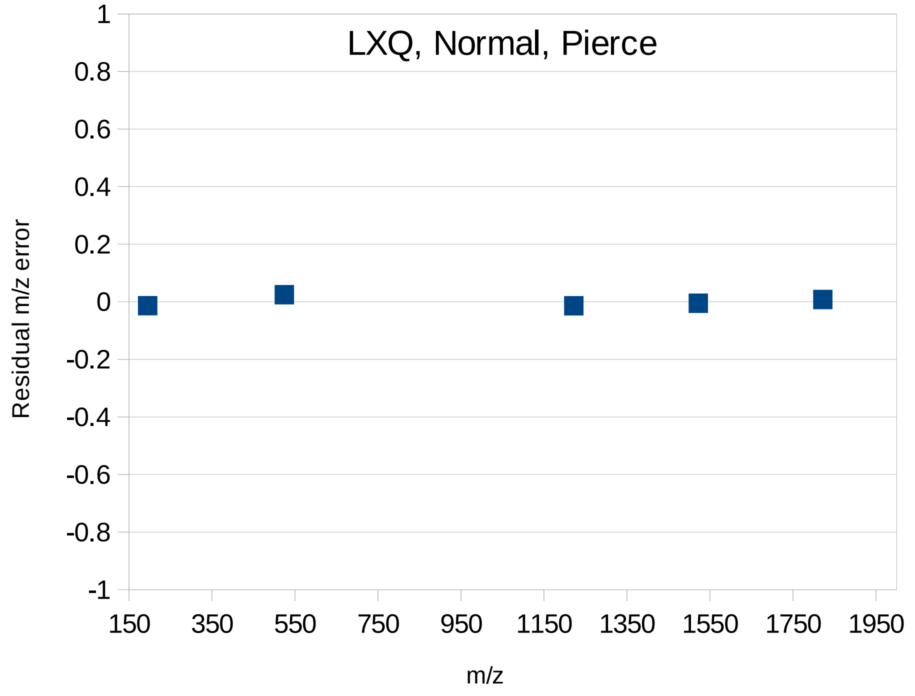
LXQ, Normal, Pierce
Residual m/z error
m/z
1
0.8
0.6
0.4
0.2
0
-0.2
-0.4
-0.6
-0.8
-1
150 350 550 750 950 1150 1350 1550 1750 1950

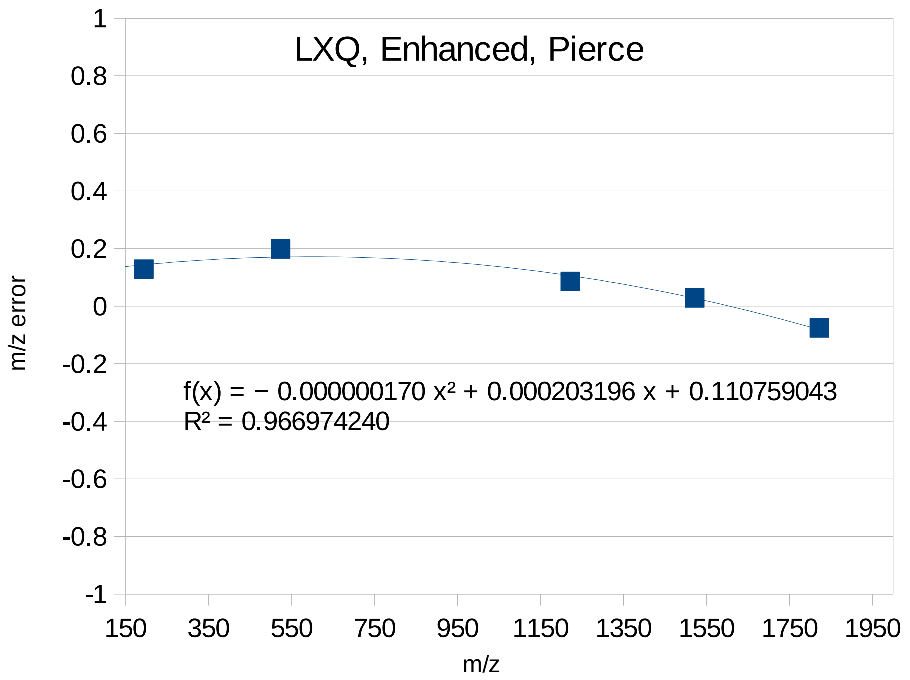
LXQ, Enhanced, Pierce
f(x) = − 0.000000170 x² + 0.000203196 x + 0.110759043
R² = 0.966974240
m/z error
m/z
1
0.8
0.6
0.4
0.2
0
-0.2
-0.4
-0.6
-0.8
-1
150
350
550
750
950
1150
1350
1550
1750
1950

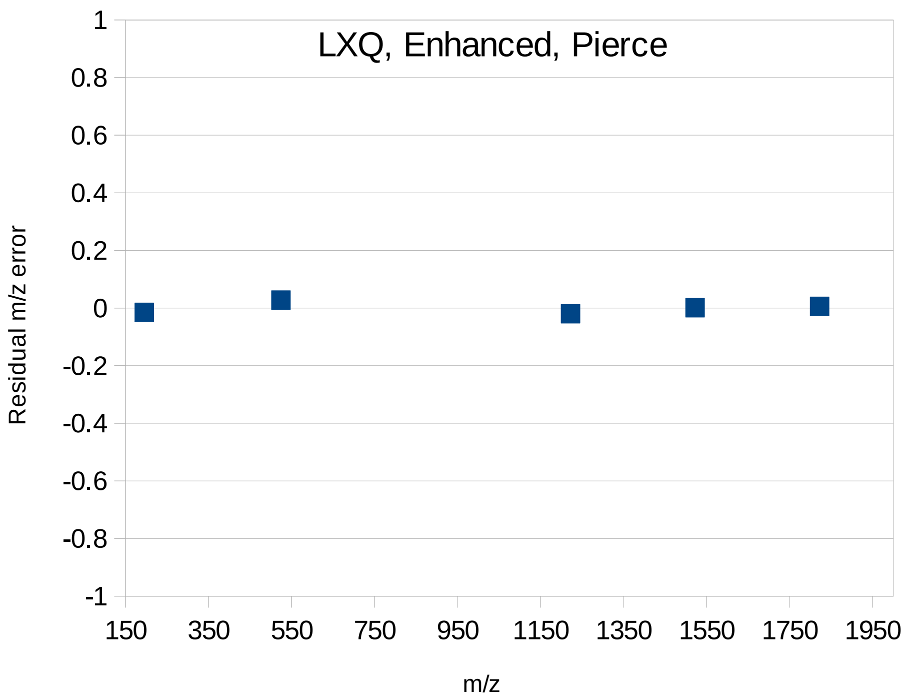
LXQ, Enhanced, Pierce
Residual m/z error
m/z
1
0.8
0.6
0.4
0.2
0
-0.2
-0.4
-0.6
-0.8
-1
150
350
550
750
950
1150
1350
1550
1750
1950

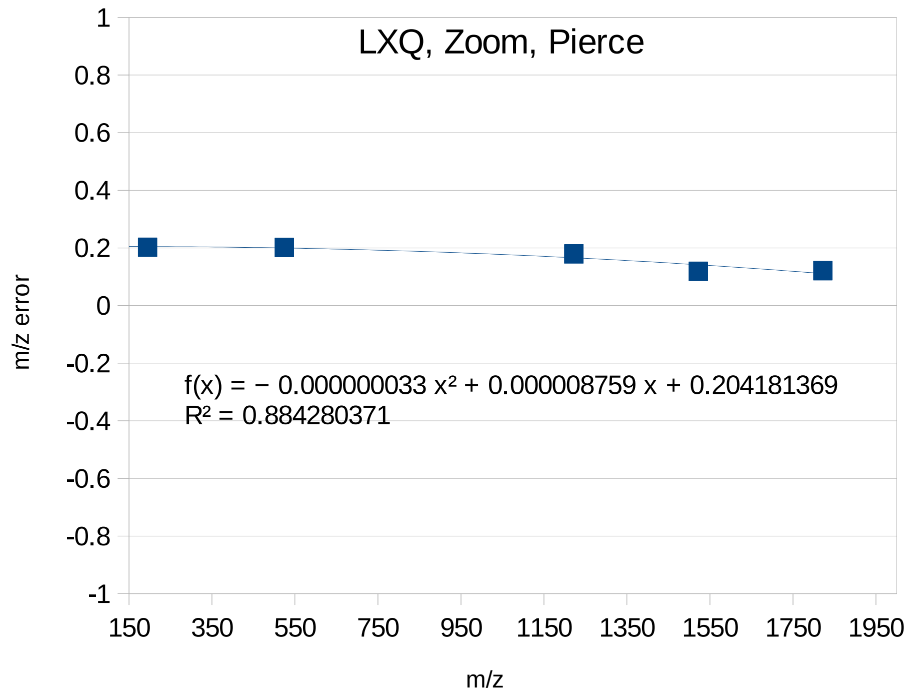
LXQ, Zoom, Pierce
f(x) = − 0.000000033 x² + 0.000008759 x + 0.204181369
R² = 0.884280371
m/z error
m/z
1
0.8
0.6
0.4
0.2
0
-0.2
-0.4
-0.6
-0.8
-1
150
350
550
750
950
1150
1350
1550
1750
1950

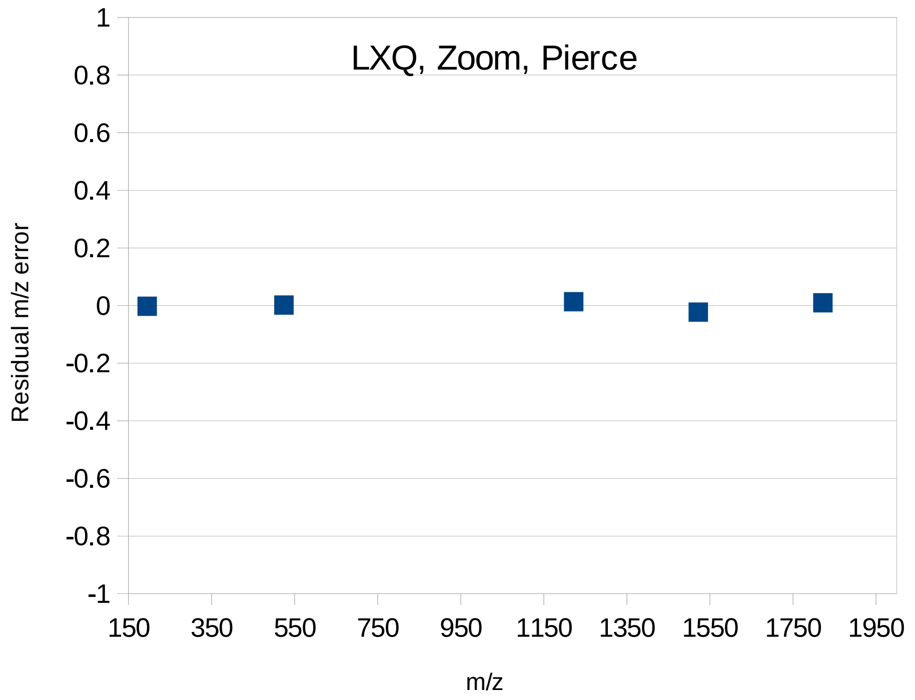
LXQ, Zoom, Pierce
Residual m/z error
m/z
1
0.8
0.6
0.4
0.2
0
-0.2
-0.4
-0.6
-0.8
-1
150
350
550
750
950
1150
1350
1550
1750
1950

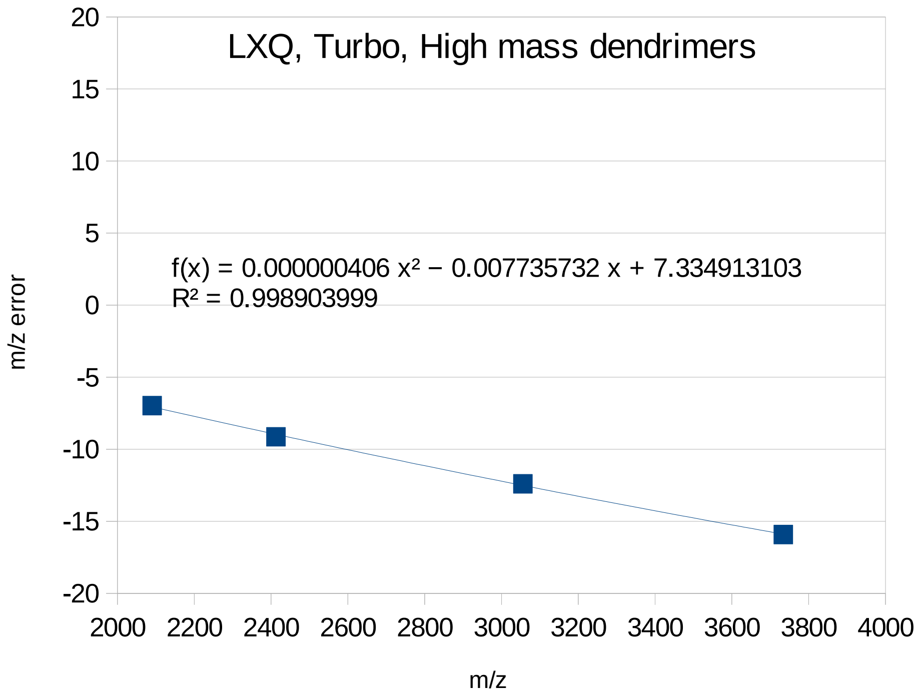
LXQ, Turbo, High mass dendrimers
f(x) = 0.000000406 x² − 0.007735732 x + 7.334913103
R² = 0.998903999
m/z error
m/z
20
15
10
5
0
-5
-10
-15
-20
2000
2200
2400
2600
2800
3000
3200
3400
3600
3800
4000

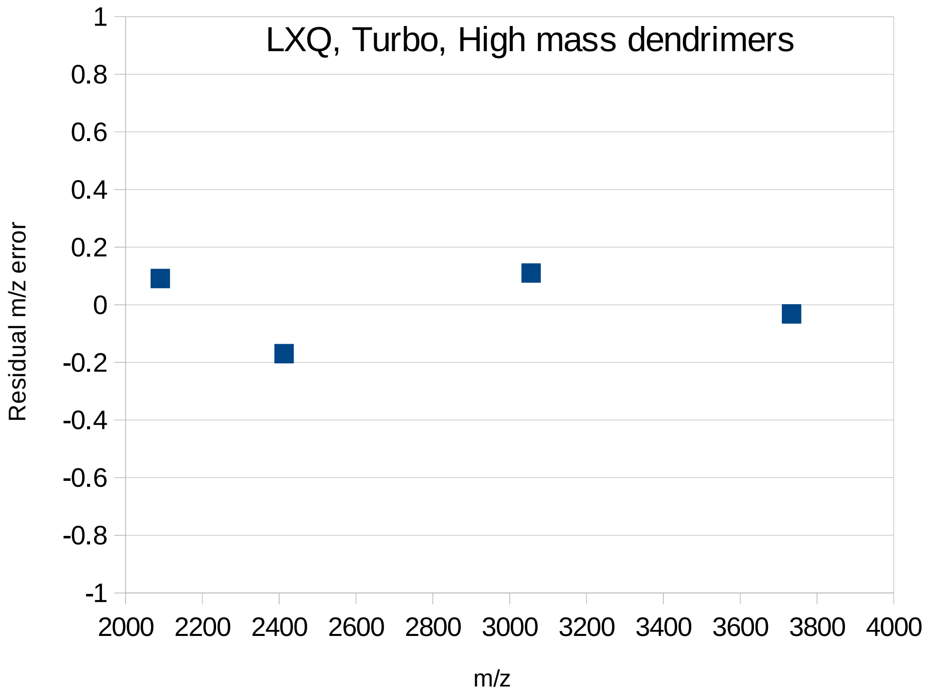
LXQ, Turbo, High mass dendrimers
Residual m/z error
m/z
1
0.8
0.6
0.4
0.2
0
-0.2
-0.4
-0.6
-0.8
-1
2000
2200
2400
2600
2800
3000
3200
3400
3600
3800
4000

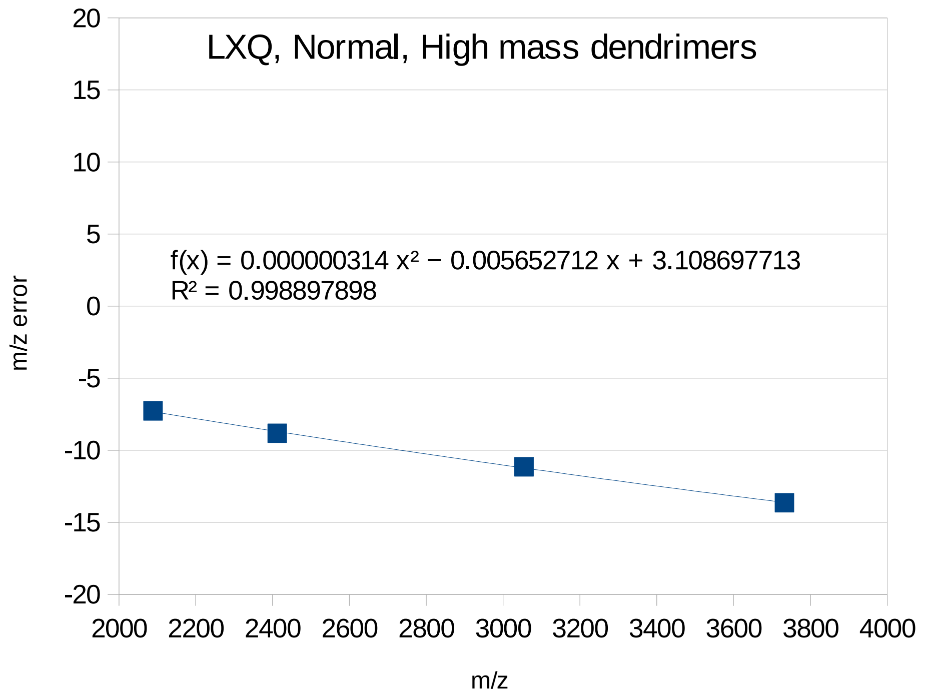

LXQ, Normal, High mass dendrimers
f(x) = 0.000000314 x² − 0.005652712 x + 3.108697713
R² = 0.998897898
m/z error
m/z
20
15
10
5
0
-5
-10
-15
-20
2000 2200 2400 2600 2800 3000 3200 3400 3600 3800 4000

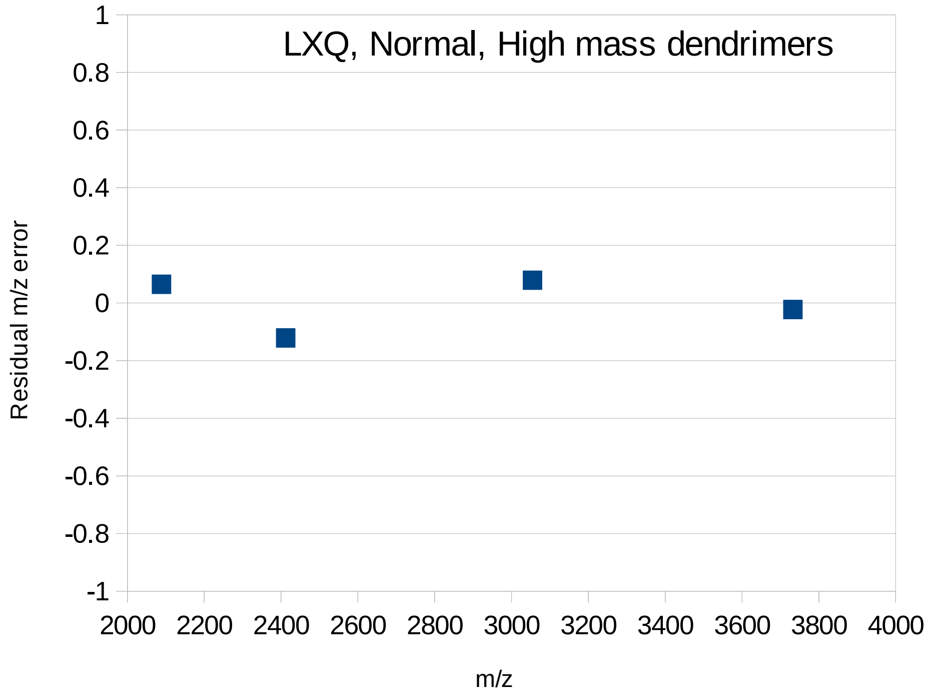

LXQ, Normal, High mass dendrimers
Residual m/z error
m/z
1
0.8
0.6
0.4
0.2
0
-0.2
-0.4
-0.6
-0.8
-1
2000 2200 2400 2600 2800 3000 3200 3400 3600 3800 4000

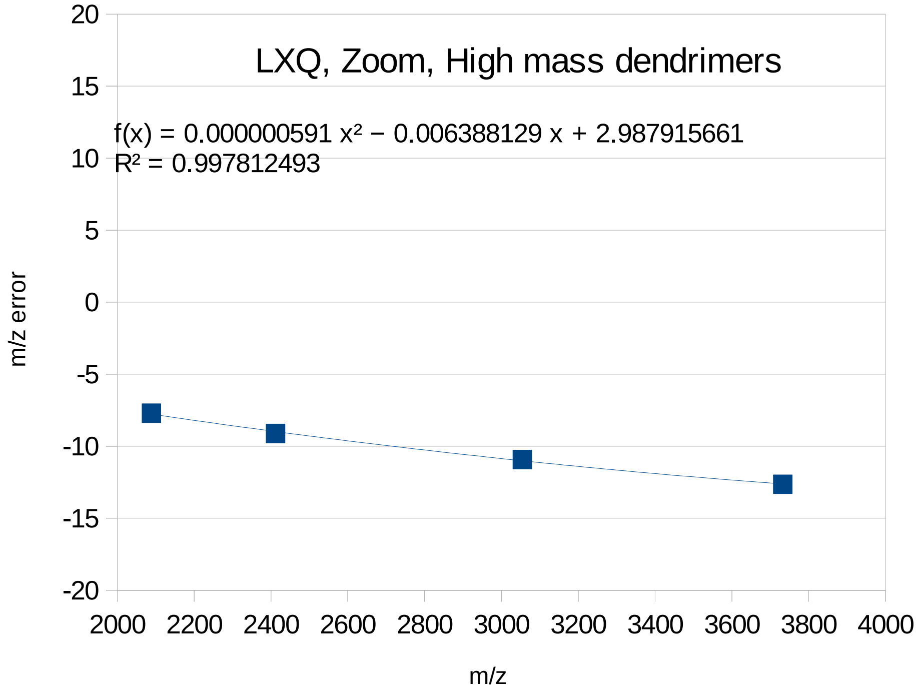
LXQ, Zoom, High mass dendrimers
f(x) = 0.000000591 x² − 0.006388129 x + 2.987915661
R² = 0.997812493
m/z error
20
15
10
5
0
-5
-10
-15
-20
2000
2200
2400
2600
2800
3000
3200
3400
3600
3800
4000
m/z

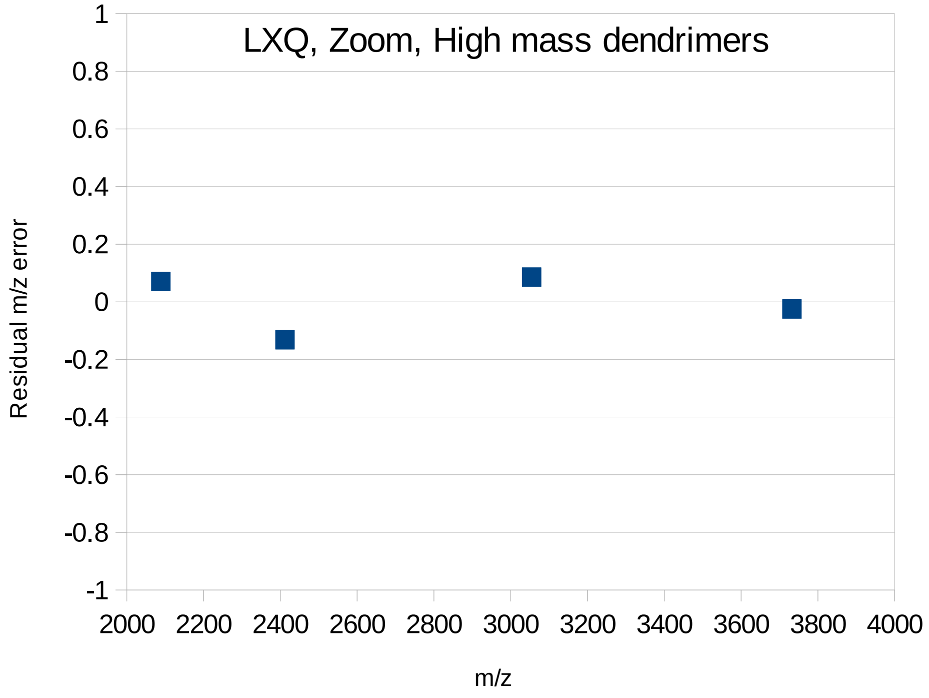
LXQ, Zoom, High mass dendrimers
Residual m/z error
1
0.8
0.6
0.4
0.2
0
-0.2
-0.4
-0.6
-0.8
-1
2000
2200
2400
2600
2800
3000
3200
3400
3600
3800
4000
m/z

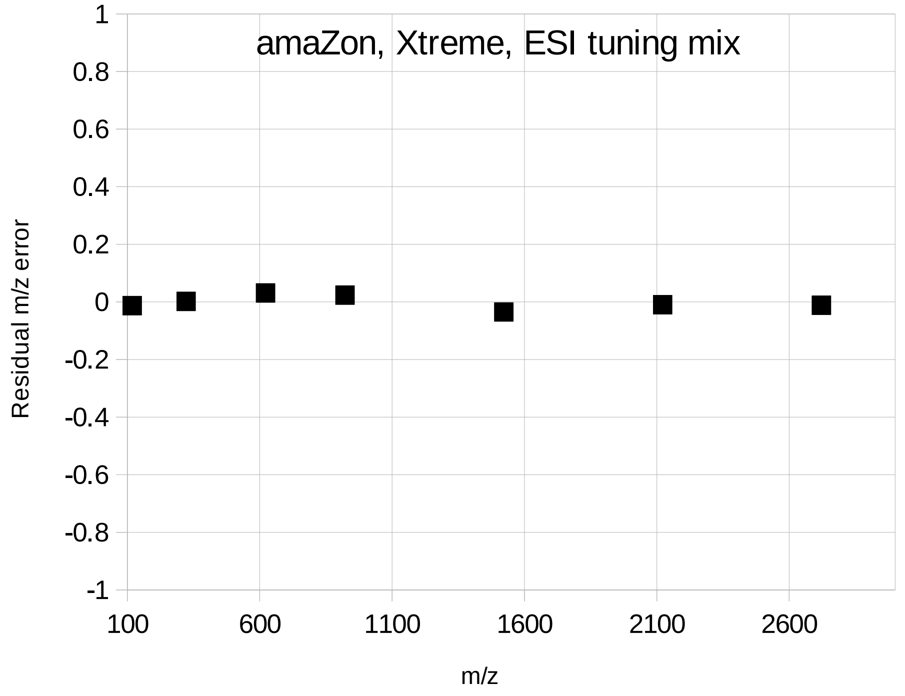
amaZon, Xtreme, ESI tuning mix
Residual m/z error
1
0.8
0.6
0.4
0.2
0
-0.2
-0.4
-0.6
-0.8
-1
100
600
1100
1600
2100
2600
m/z

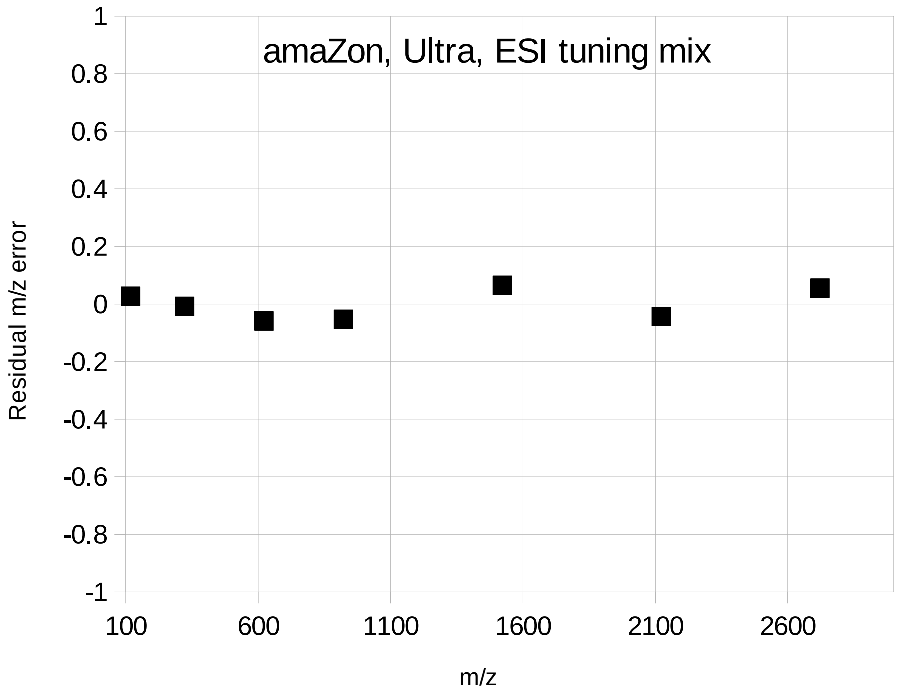
amaZon, Ultra, ESI tuning mix
Residual m/z error
m/z
1
0.8
0.6
0.4
0.2
0
-0.2
-0.4
-0.6
-0.8
-1
100
600
1100
1600
2100
2600

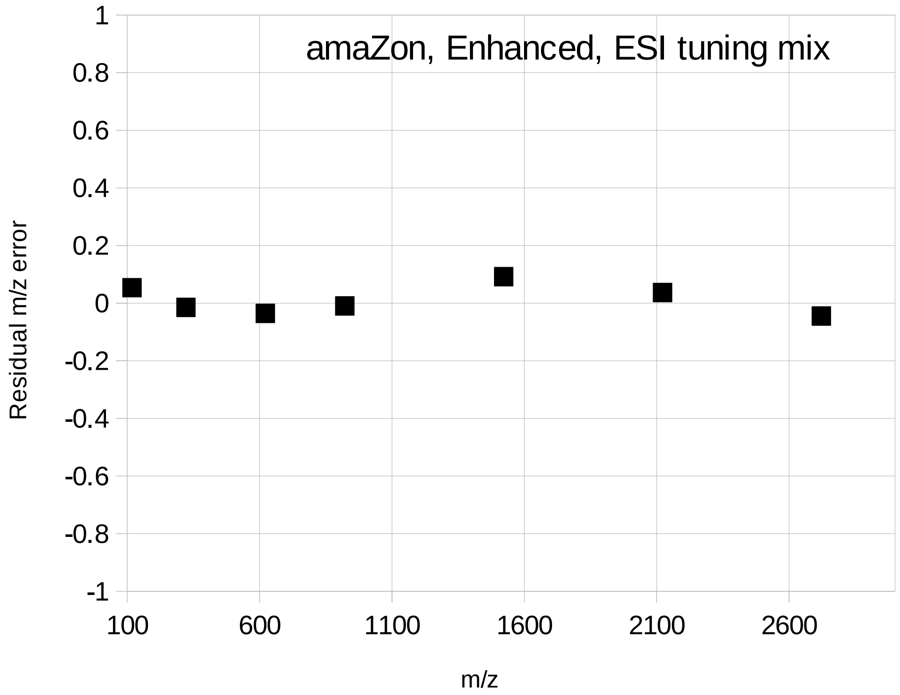
amaZon, Enhanced, ESI tuning mix
Residual m/z error
m/z
1
0.8
0.6
0.4
0.2
0
-0.2
-0.4
-0.6
-0.8
-1
100
600
1100
1600
2100
2600

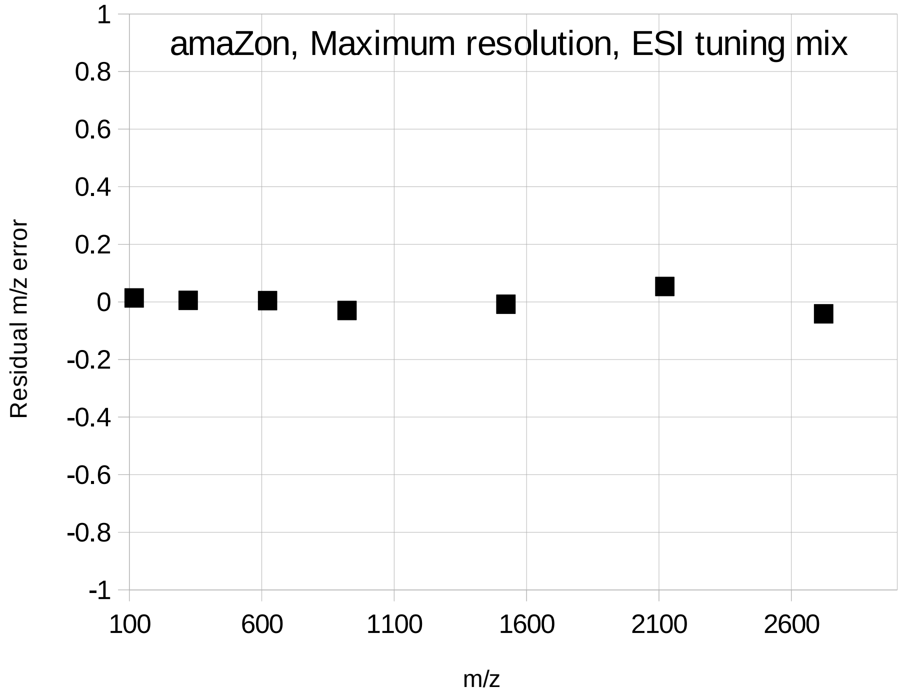
amaZon, Maximum resolution, ESI tuning mix
Residual m/z error
1
0.8
0.6
0.4
0.2
0
-0.2
-0.4
-0.6
-0.8
-1
100
600
1100
1600
2100
2600
m/z

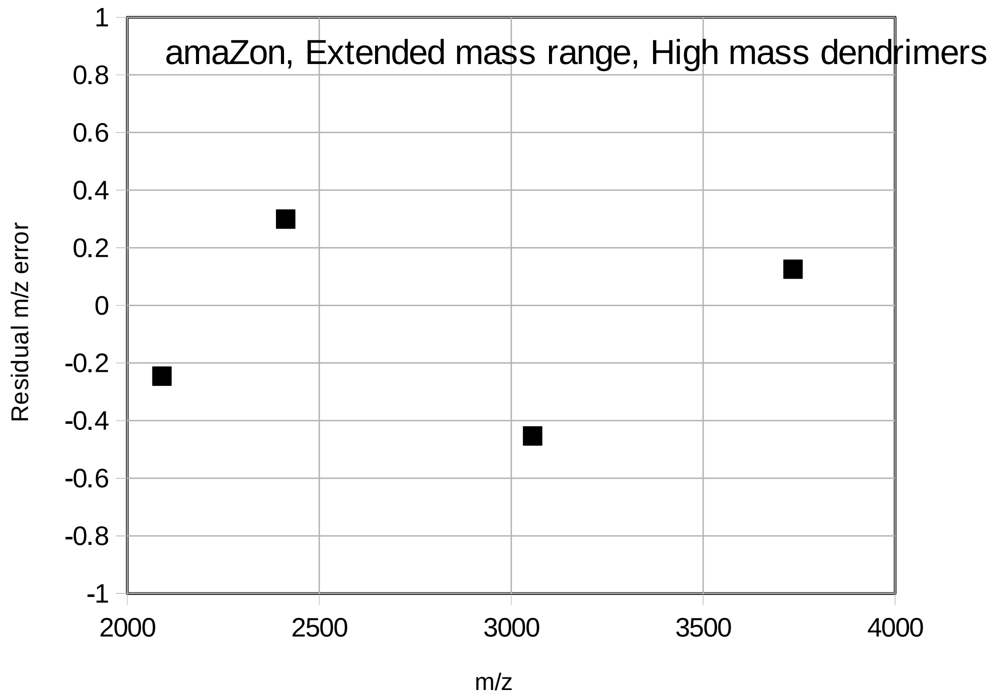
amaZon, Extended mass range, High mass dendrimers
1
0.8
0.6
0.4
0.2
0
-0.2
-0.4
-0.6
-0.8
-1
Residual m/z error
2000
2500
3000
3500
4000
m/z

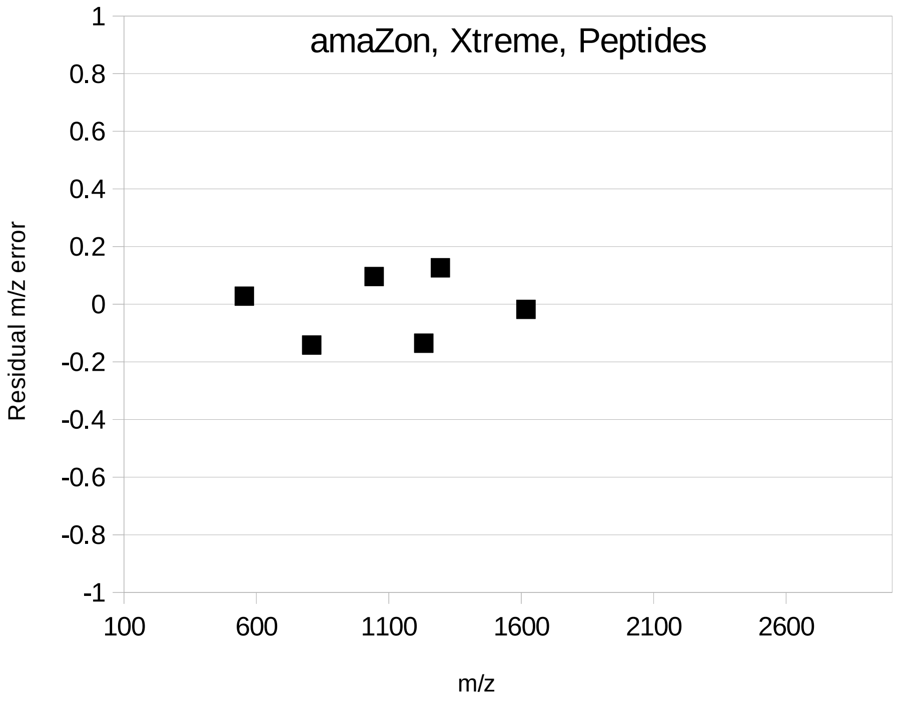
amaZon, Xtreme, Peptides
Residual m/z error
1
0.8
0.6
0.4
0.2
0
-0.2
-0.4
-0.6
-0.8
-1
100
600
1100
1600
2100
2600
m/z

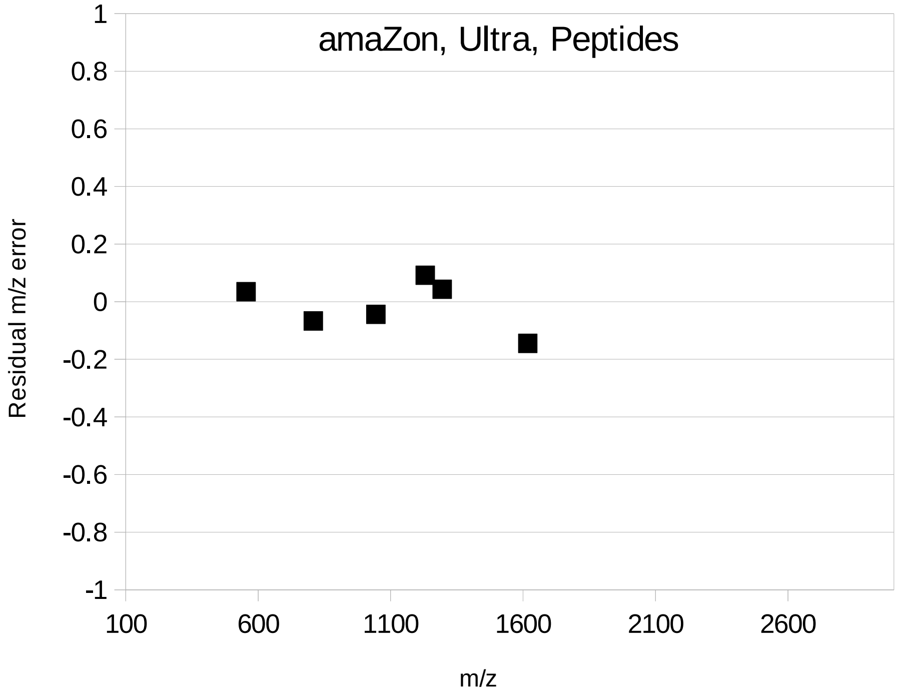
amaZon, Ultra, Peptides
Residual m/z error
m/z
1
0.8
0.6
0.4
0.2
0
-0.2
-0.4
-0.6
-0.8
-1
100
600
1100
1600
2100
2600

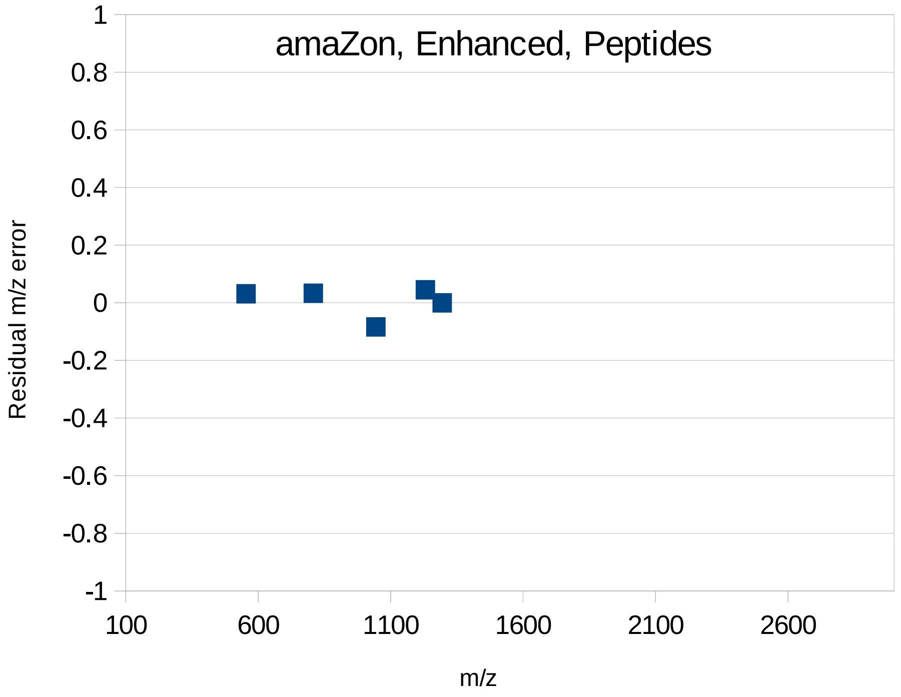
amaZon, Enhanced, Peptides
Residual m/z error
1
0.8
0.6
0.4
0.2
0
-0.2
-0.4
-0.6
-0.8
-1
100
600
1100
1600
2100
2600
m/z

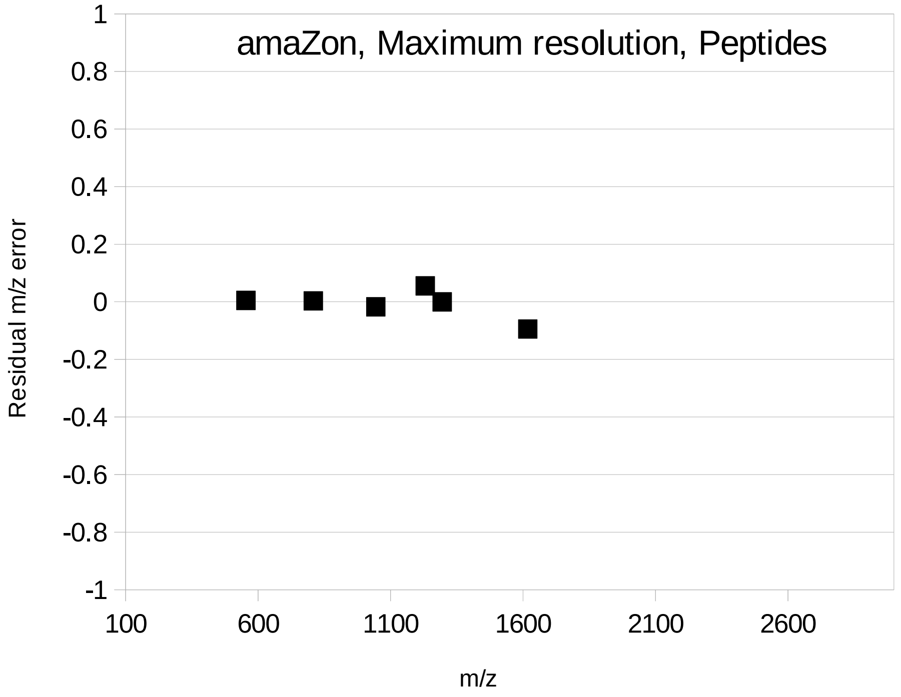
amaZon, Maximum resolution, Peptides
Residual m/z error
1
0.8
0.6
0.4
0.2
0
-0.2
-0.4
-0.6
-0.8
-1
100
600
1100
1600
2100
2600
m/z
