# Supplementary material for: Chemical mass shifts of cluster ions and adduct ions in quadrupolar ion traps revisited and extended
Source: Rapid Commun Mass Spectrom. 2022 Dec 14;37(3):e9436. doi: 10.1002/rcm.9436 (PMC10078176; doi:10.1002/rcm.9436)
Supplement: Supplementary file 2 — DATA S2 Supplementary mass shifts [file RCM-37-0-s002.pdf]

Supplementary - mass shifts

m/z errors for the analyzes not presented in the paper.

Titles, axes, and legends of the charts are self-explanatory. Each datapoint is the average of three spectra, with error bars showing one standard deviation. To better fit each analysis, x-axes and y-axes do not always show the same range. Due to severe peak distortion in the amaZon QIT, *m/z* errors of clusters and adducts should only be qualitatively interpreted at higher *m/z*. Some of these very large errors are outside the plot ranges. Note that *m/z* errors in negative mode sometimes are very large at low *m/z*, due to extrapolation from the lowest calibration point at *m/z* 554.

Table 1: List of m/z error plots

Experiment	Analytes	Charge carrier	Fig. - m/z error	
			LXQ LIT	amaZon QIT
Low mass range	Y(HCOO)3	+ $HCOO^-$		Sup. Fig. 13 - 20
	NaCF3COO	+ Na^+		Sup. Fig. 5 - 12
	ESI Tuning mix	+ H^+		Sup. Fig. 5 - 12
		+ $CF3COO^-$		Sup. Fig. 13 - 18
	Spherical-ESI	+ Na^+		Sup. Fig. 5 - 12
	Peptides	+ H^+		Sup. Fig. 5 - 12
		- H^+		Sup. Fig. 13 - 20
	Pierce calibration mixture	+ H^+	Sup. Fig. 1, 2, 3, 4	
	CsI	+ Cs^+	Sup. Fig. 1, 2, 3, 4	Sup. Fig. 5 - 12
		+ I^-		Sup. Fig. 13 - 20
	Pierce calibration mixture<br>+<br>CsI	+ H^+<br>+ Cs^+	Fig. 1, column 1	Fig. 1, column 2
	High mass dendrimer mixture	+ Na^+		Sup. Fig. 5 - 12
		+ Cs^+		Sup. Fig. 5 - 12
High mass range	High mass dendrimer mixture	+ Na^+<br>+ Cs^+	Fig. 2, column 1	Fig. 2
	CsI	+ Cs^+<br>+ 2 Cs^+	Fig. 2, column 1	Fig. 2
	ESI Tuning mix	+ H^+		Fig. 2

LXQ Linear ion trap, positive mode

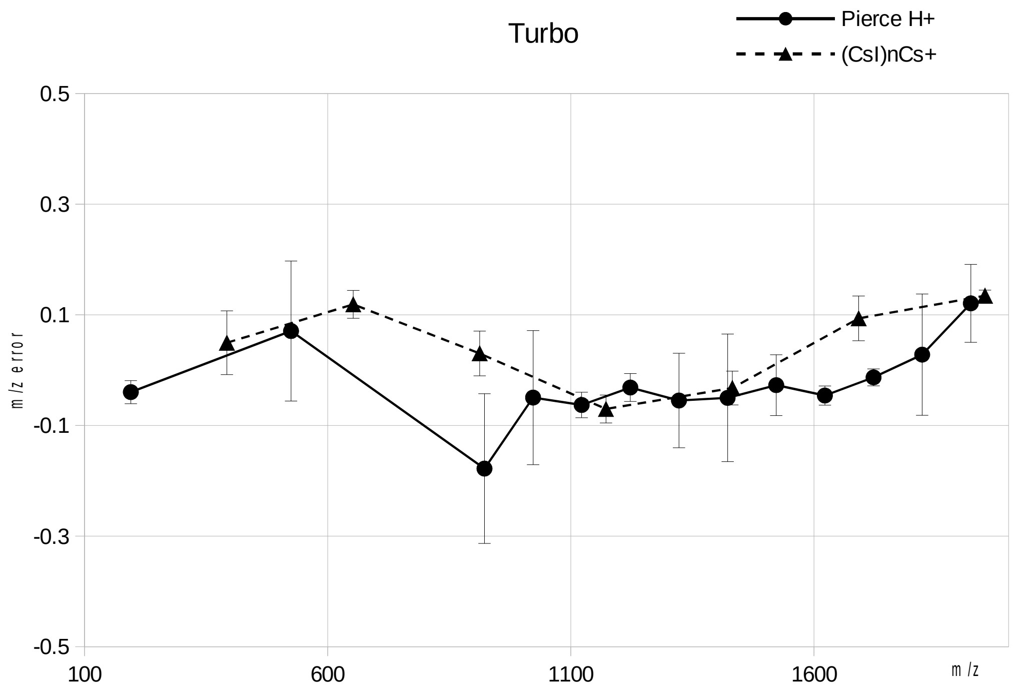

Figure 1

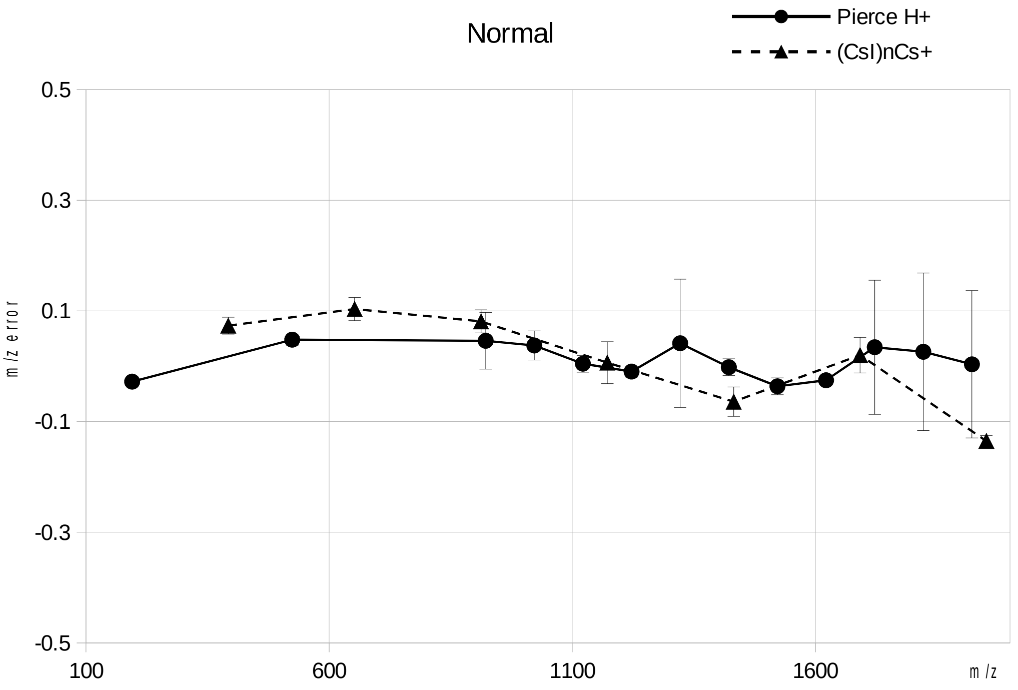

Figure 2

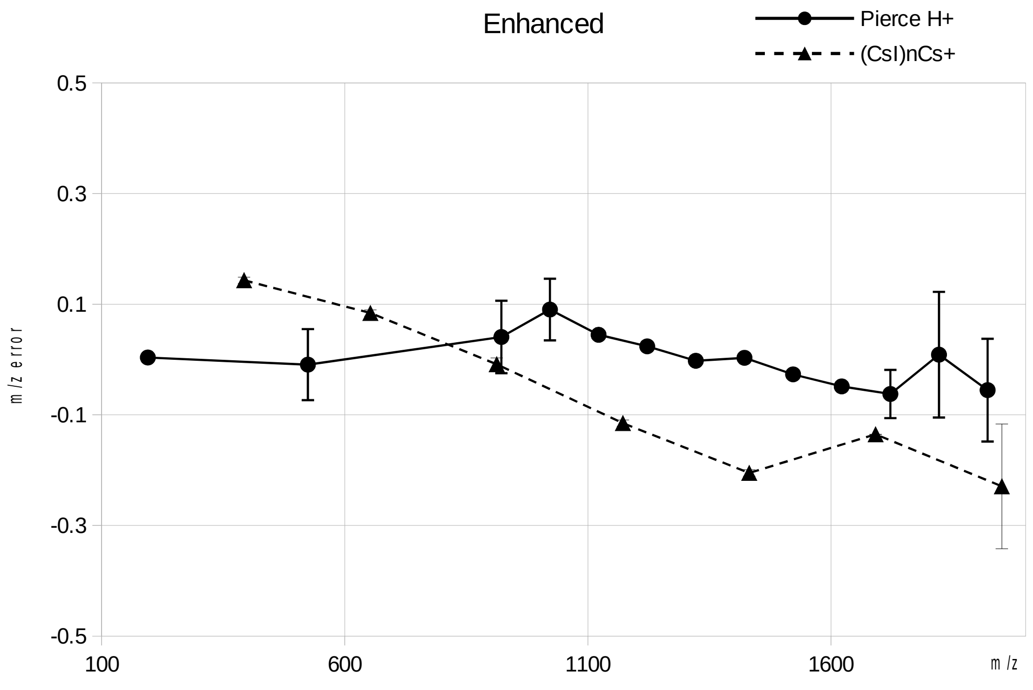

Figure 3

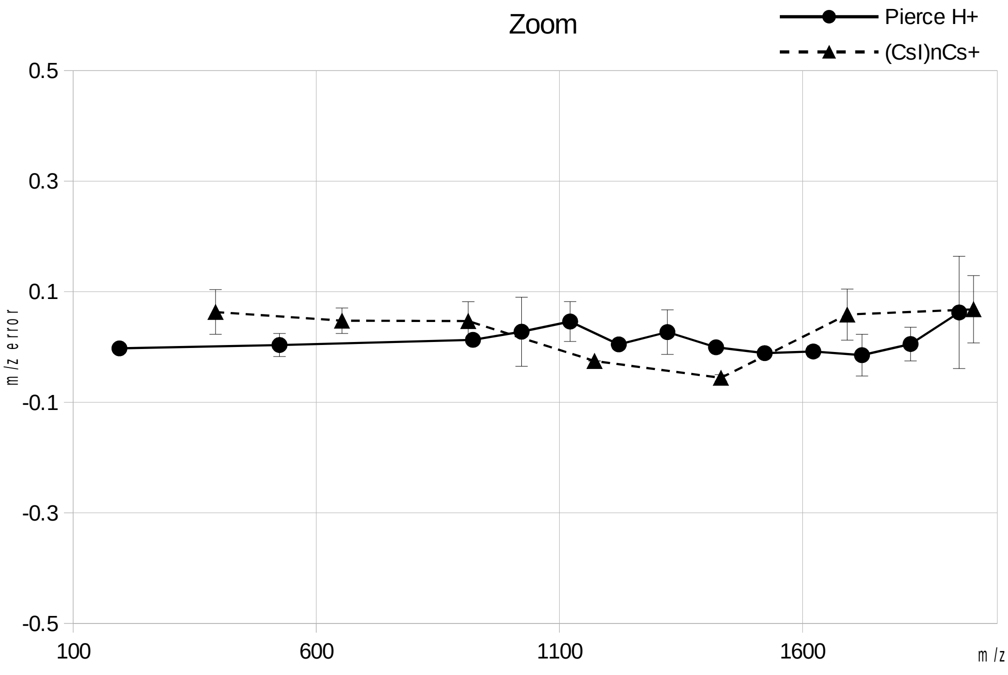

Figure 4

amaZon spherical ion trap, positive mode

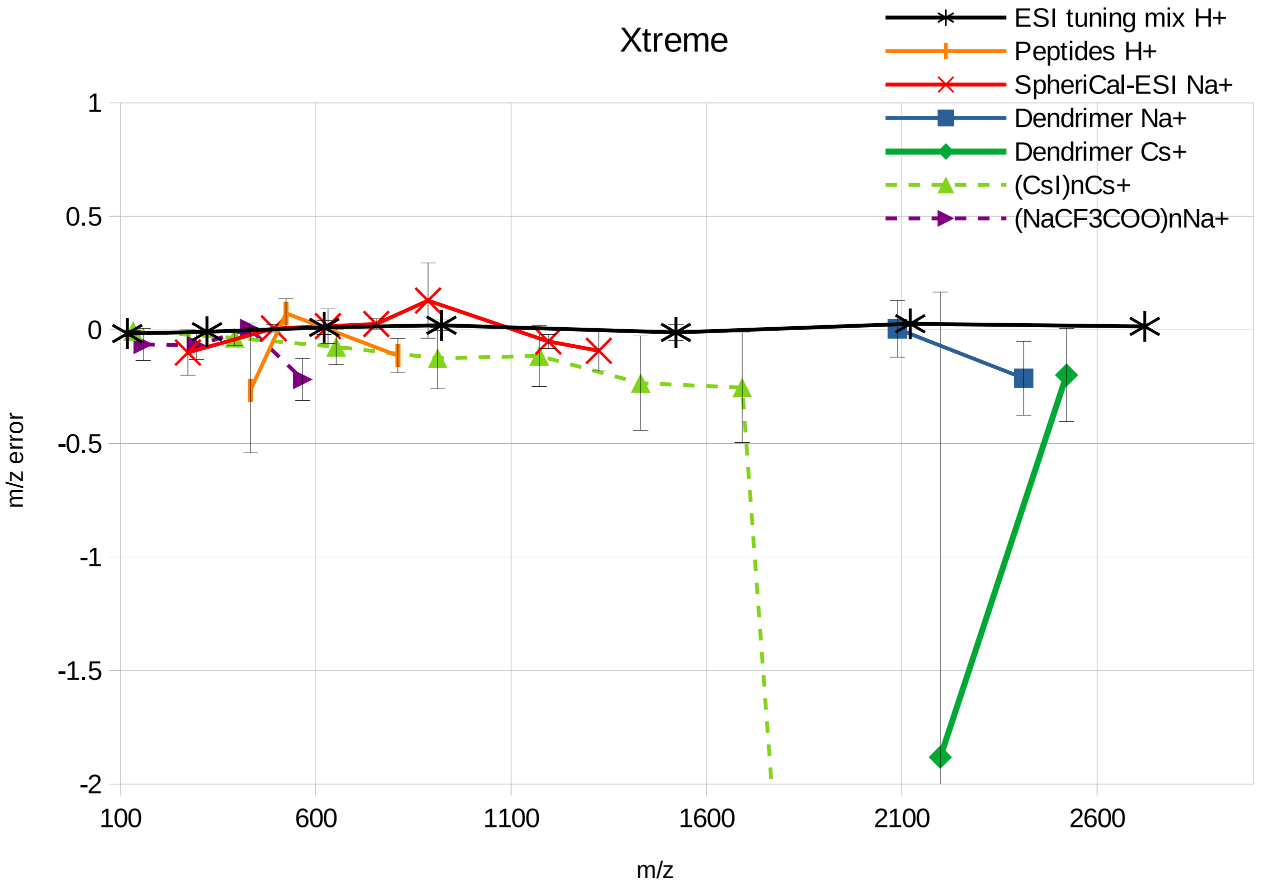

Figure 5

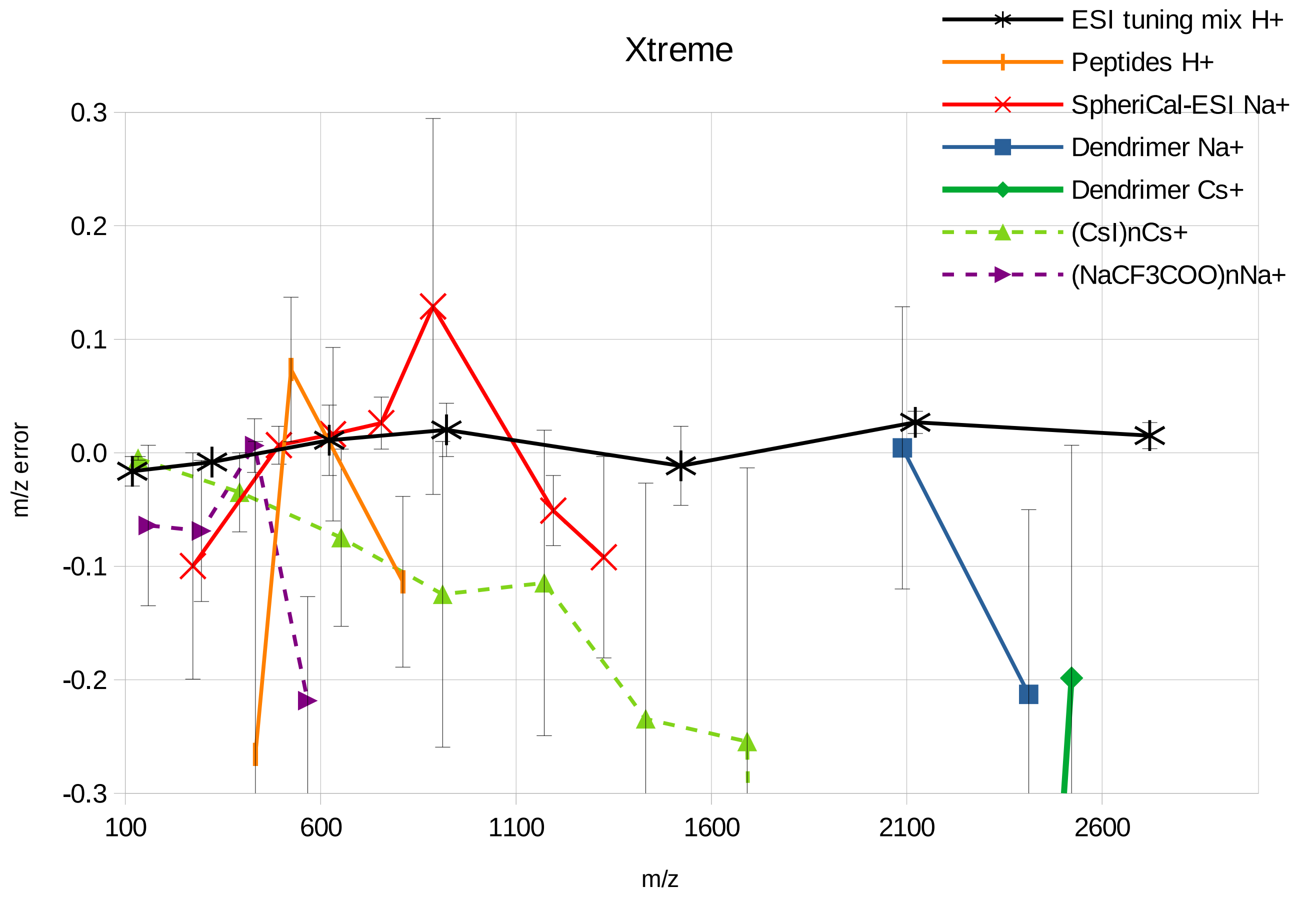

Figure 6

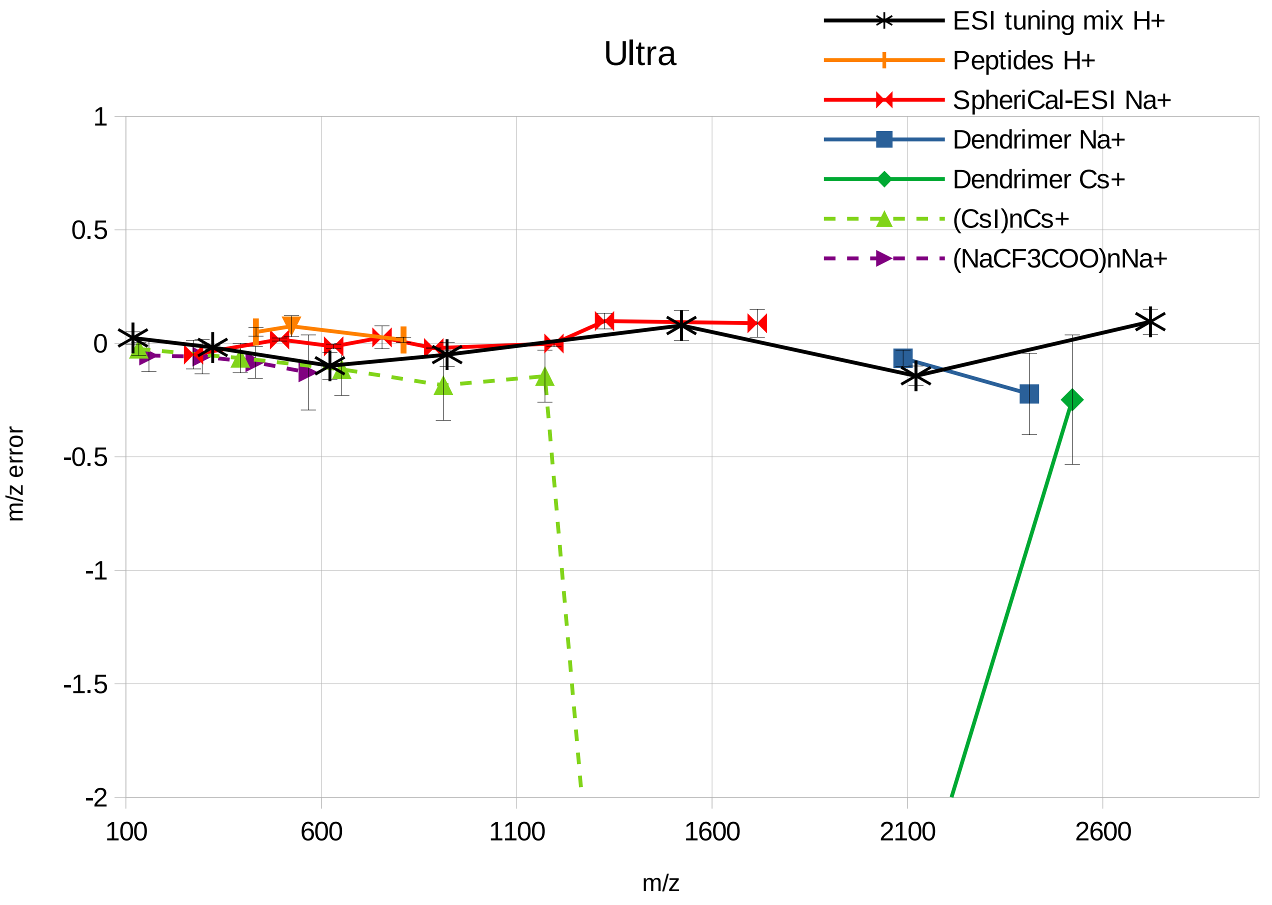

Figure 7

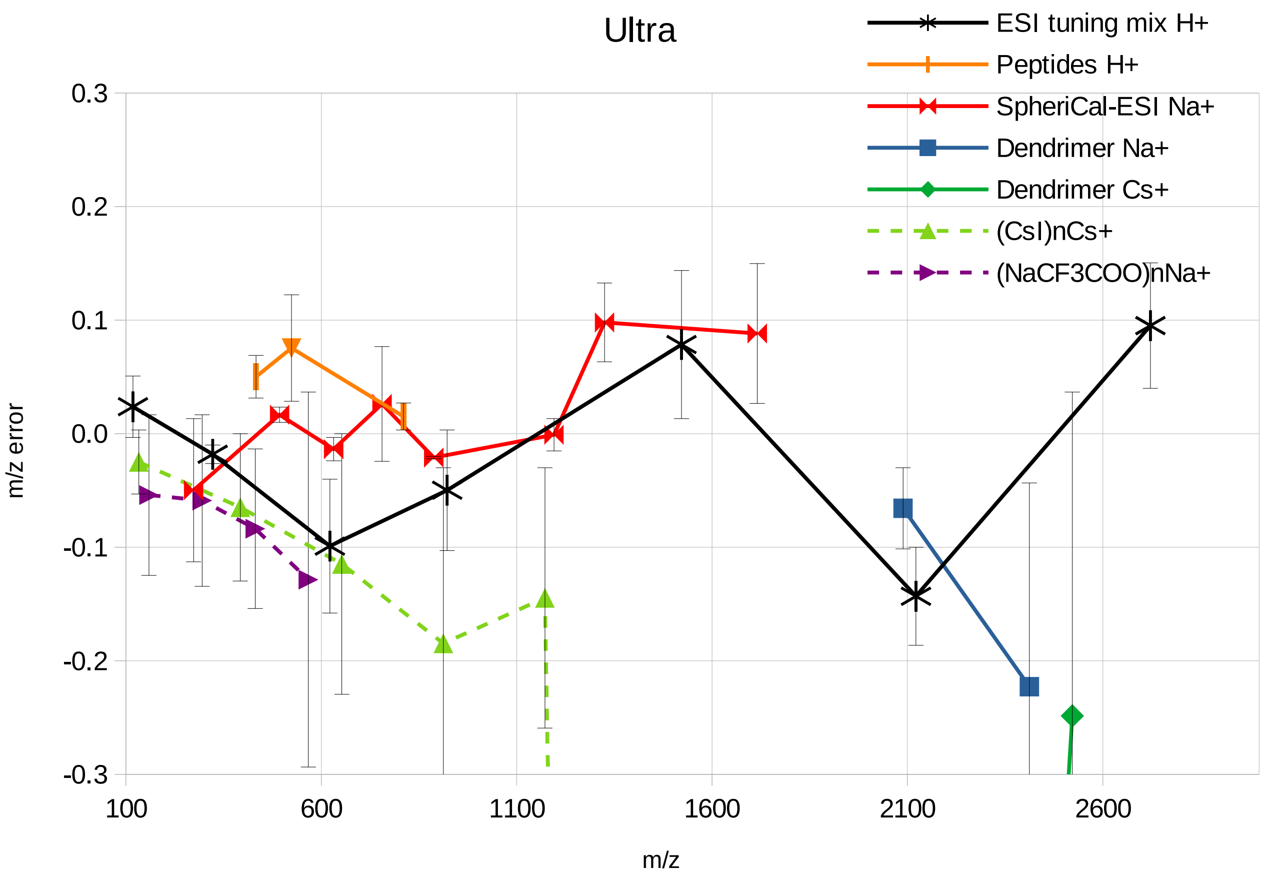

Figure 8

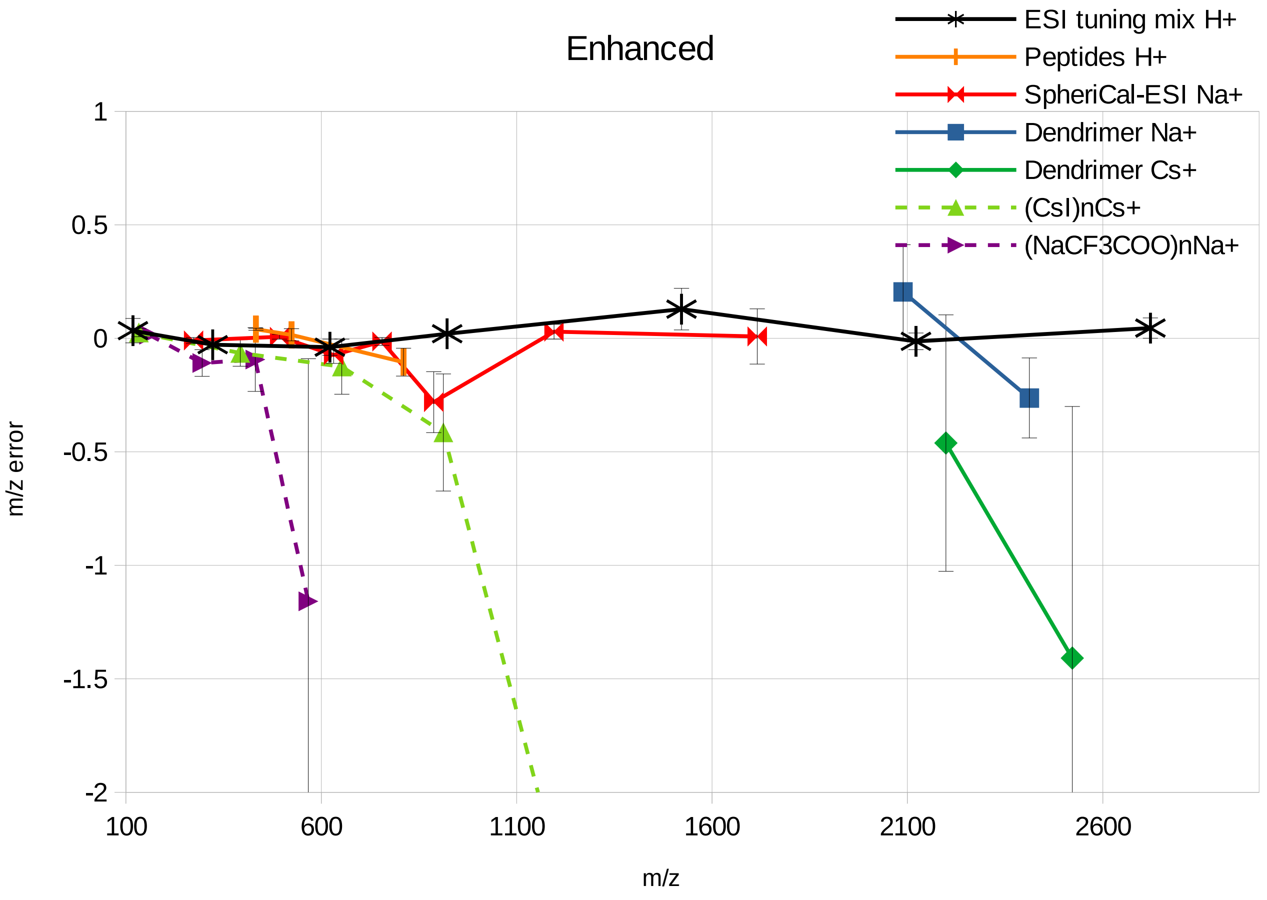

Figure 9

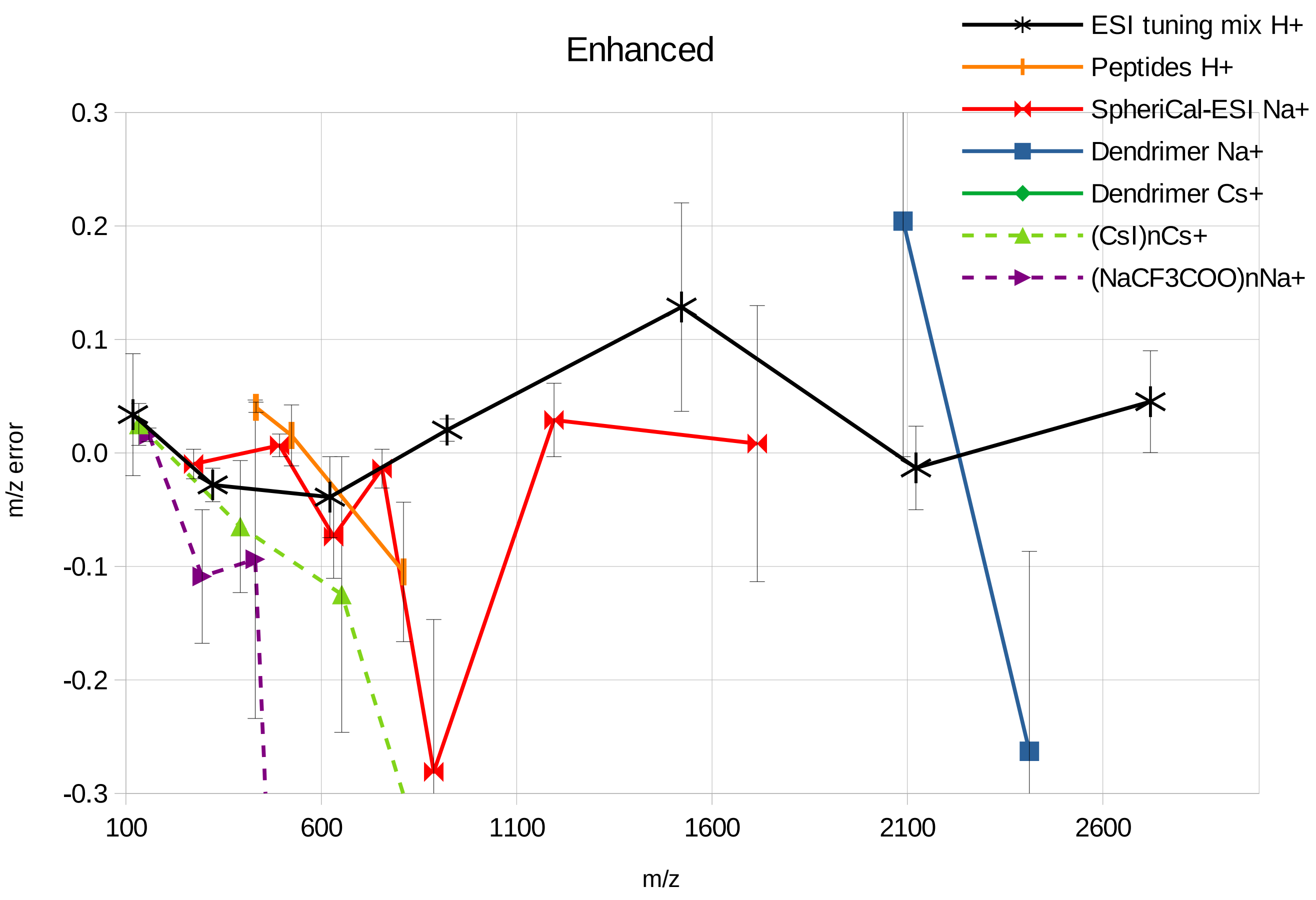

Figure 10

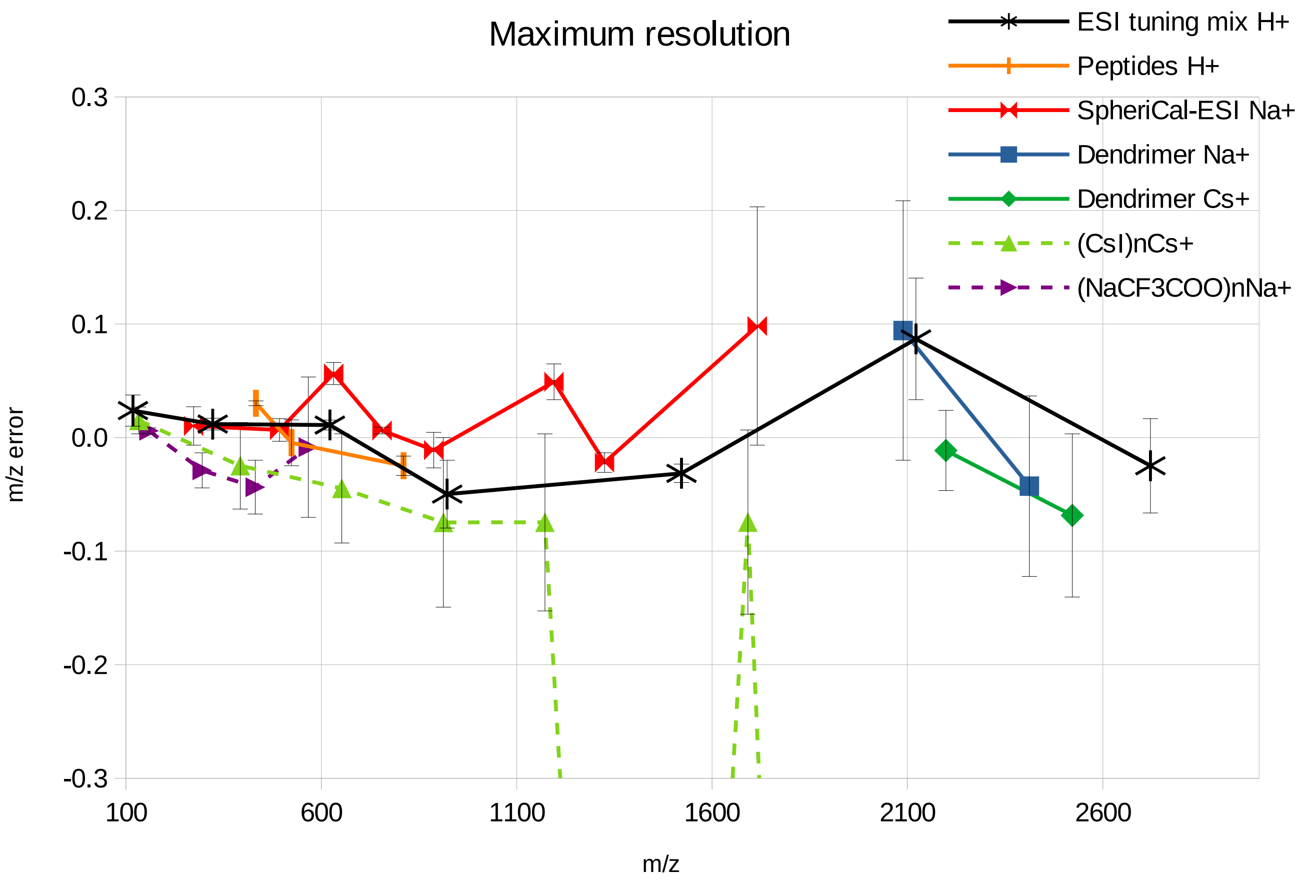

Figure 11

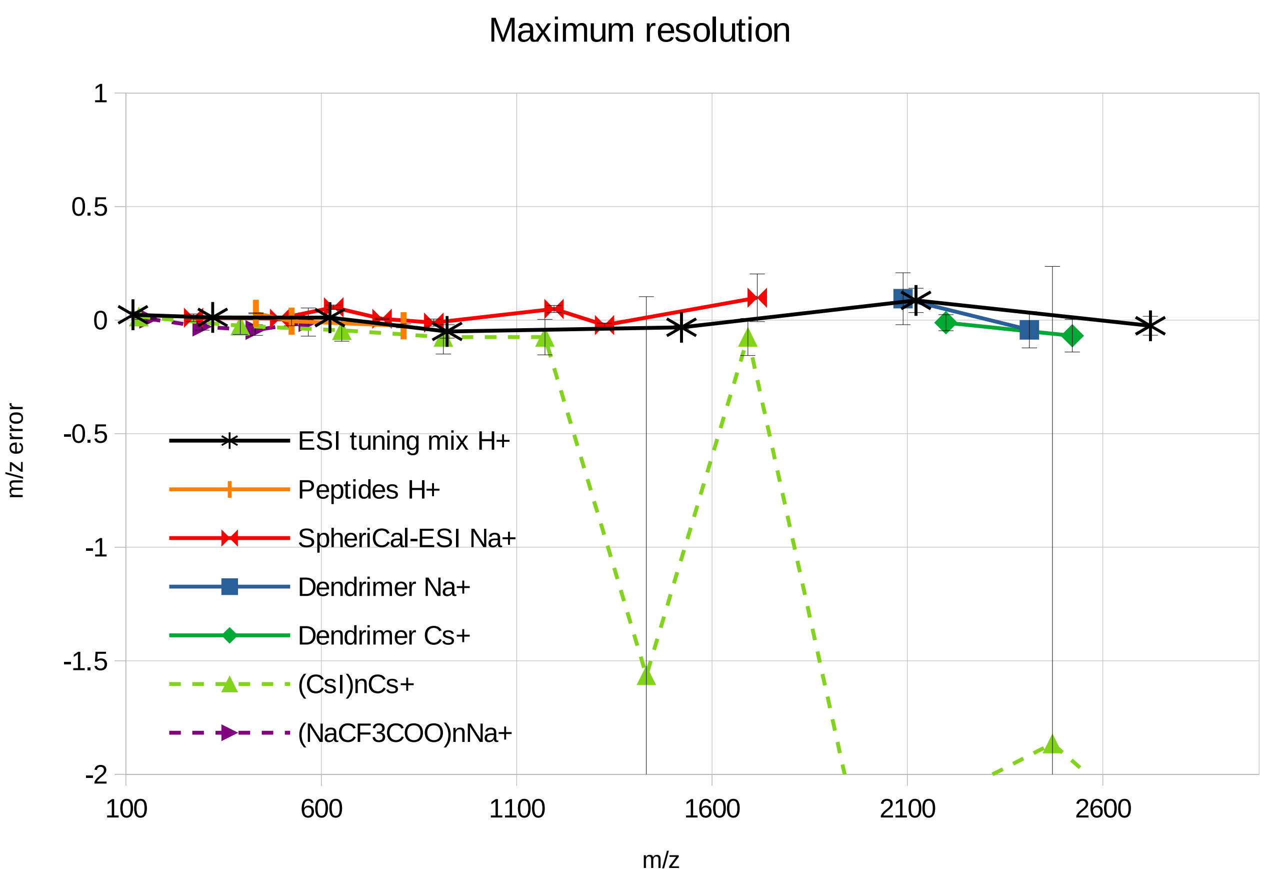

Maximum resolution
1
0.5
0
-0.5
-1
-1.5
-2
m/z error
100
600
1100
1600
2100
2600
m/z
ESI tuning mix H+
Peptides H+
SpheriCal-ESI Na+
Dendrimer Na+
Dendrimer Cs+
(CsI)nCs+
(NaCF3COO)nNa+

Figure 12

amaZon spherical ion trap, negative mode

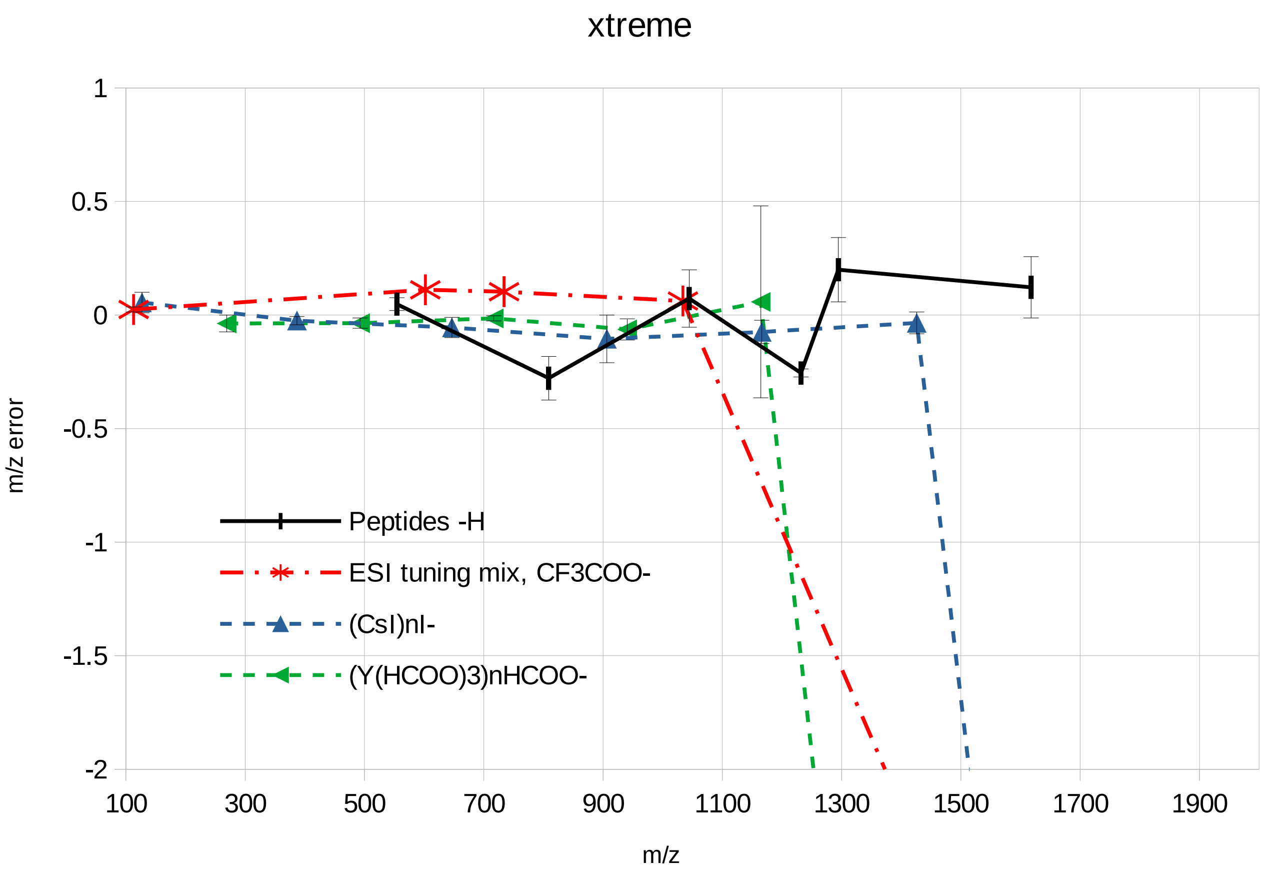

Figure 13

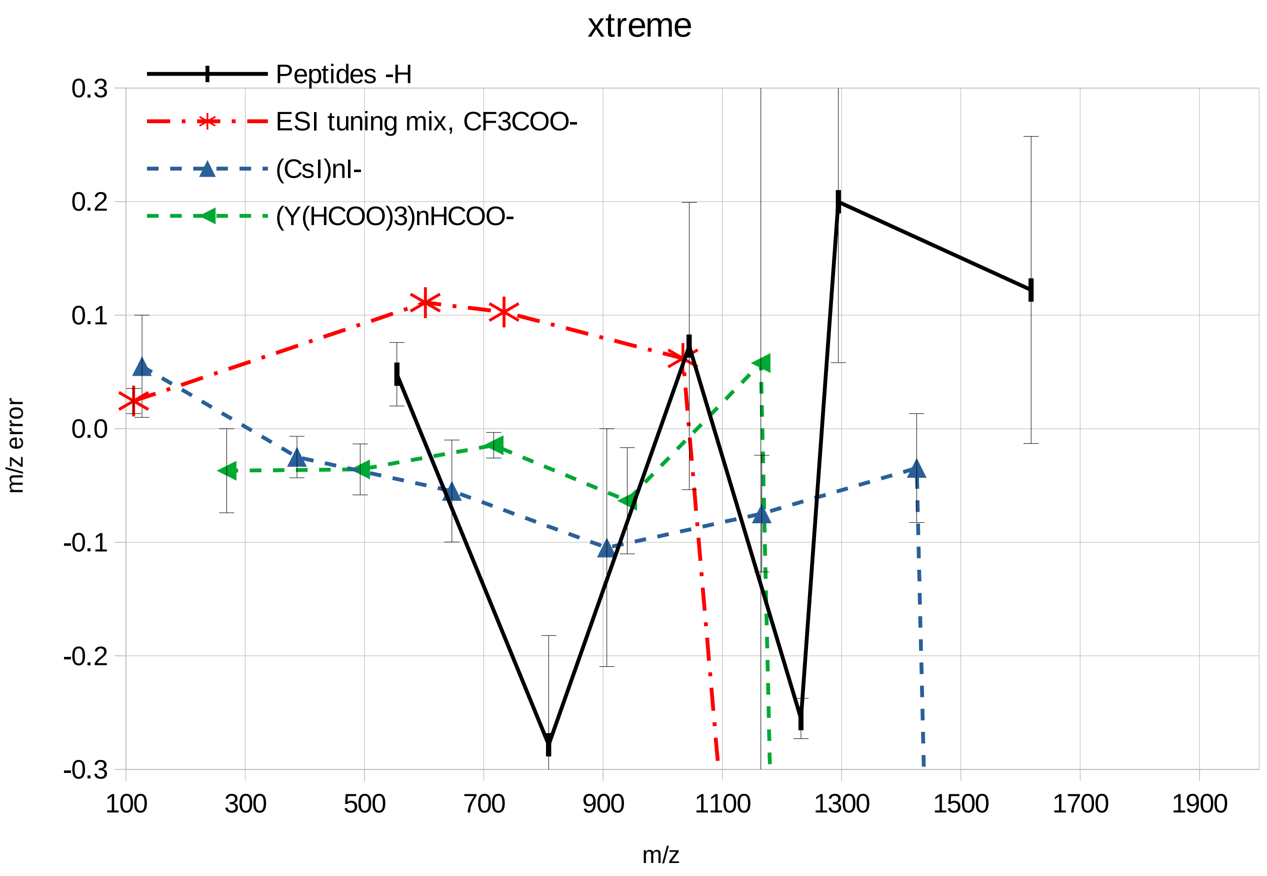

Figure 14

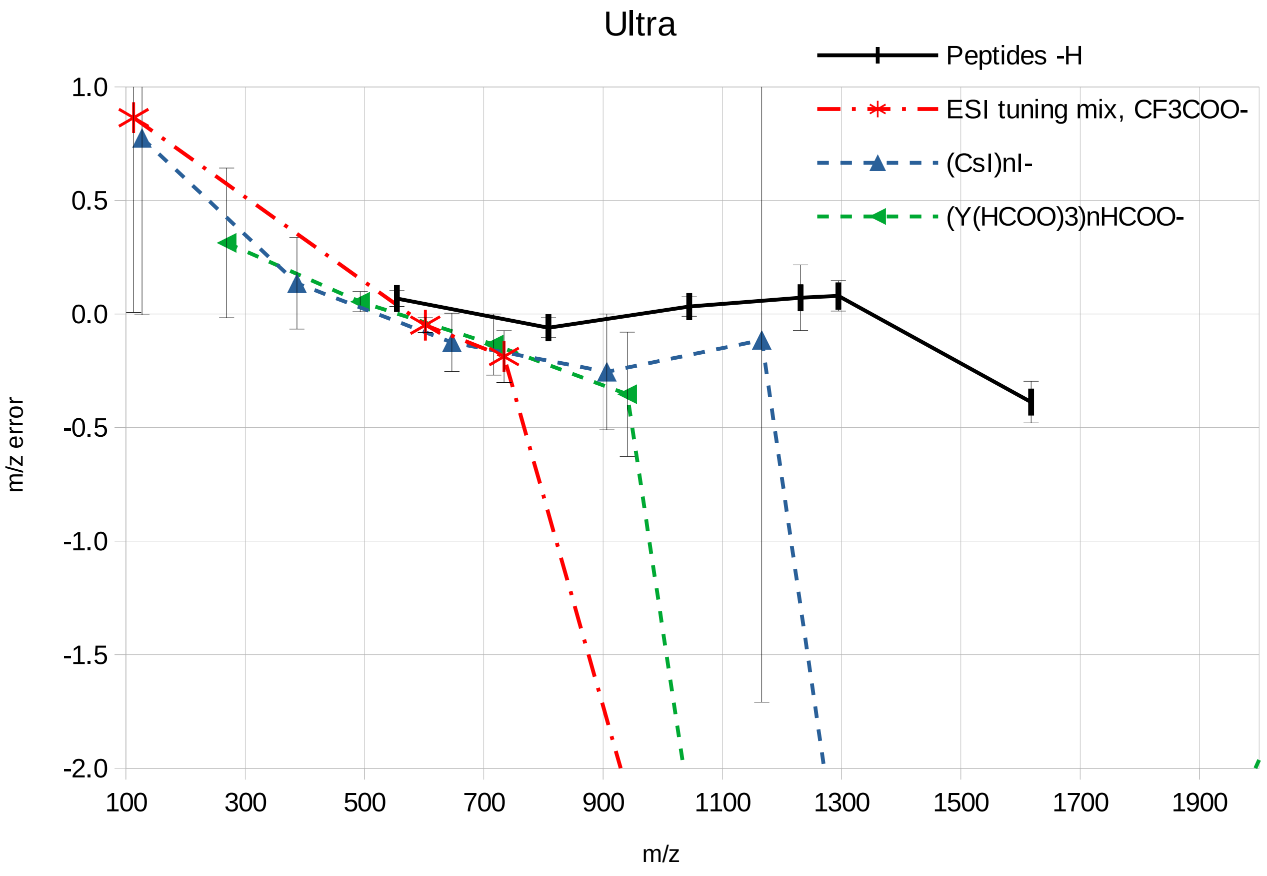

Figure 15

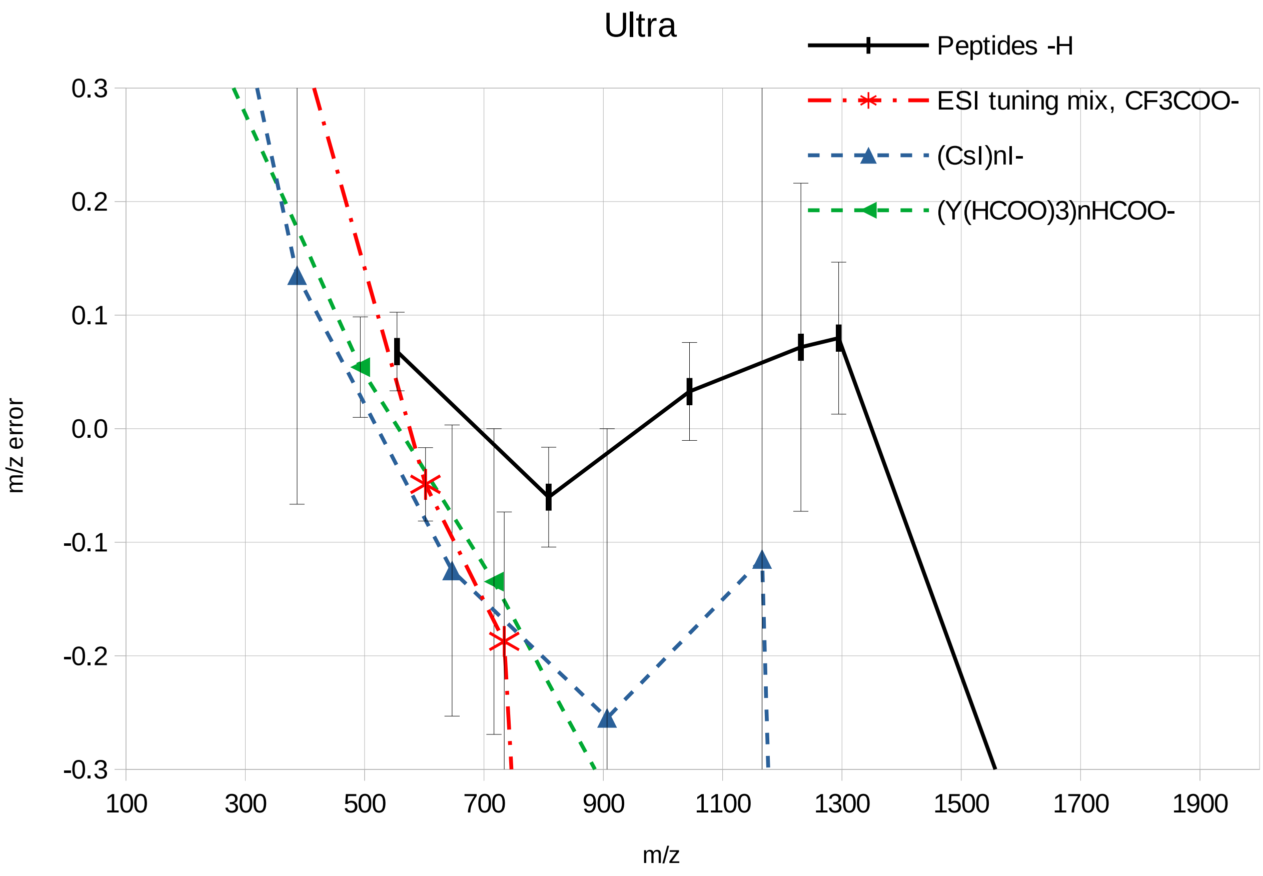

Figure 16

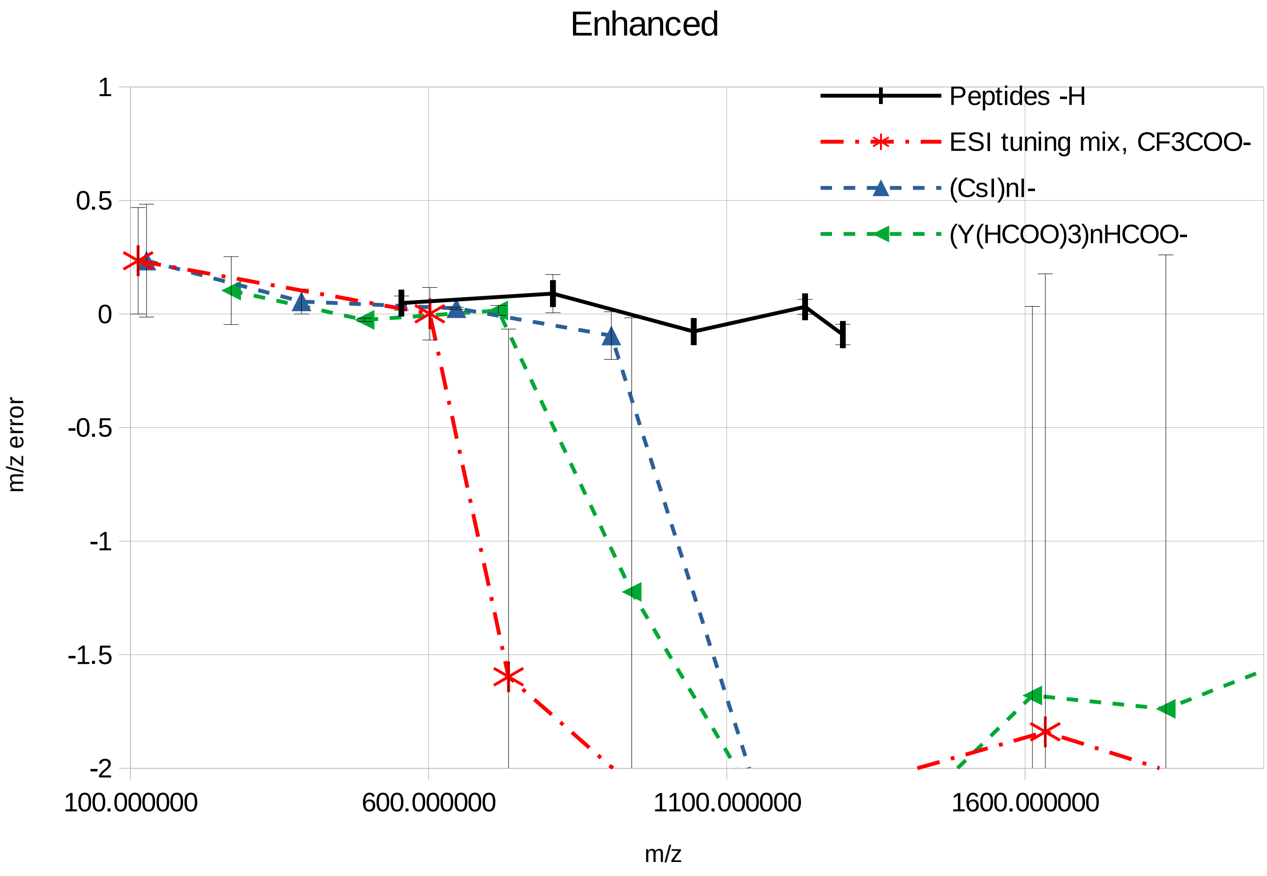

Figure 17

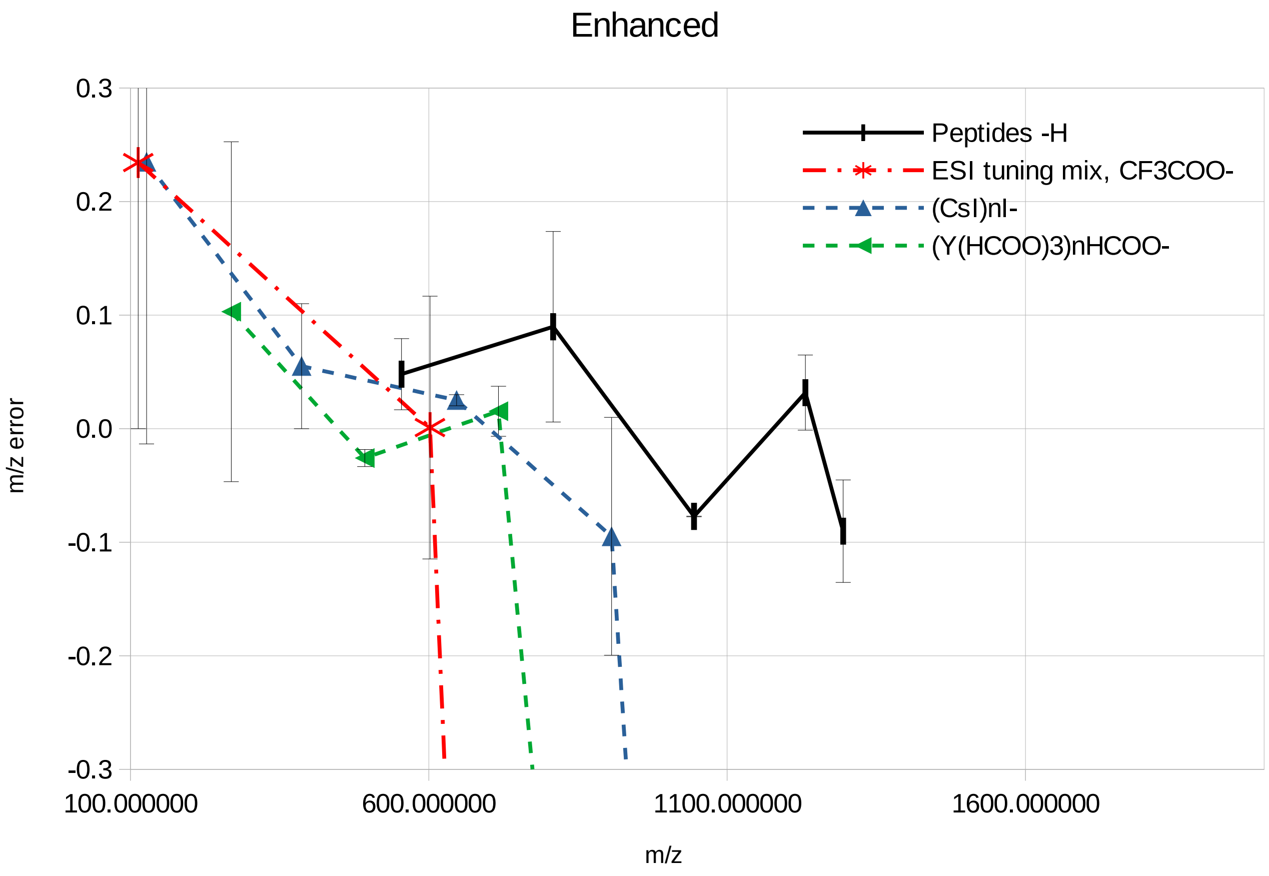
Enhanced
0.3
0.2
0.1
0.0
-0.1
-0.2
-0.3
m/z error
100.000000
600.000000
1100.000000
1600.000000
m/z
Peptides -H
ESI tuning mix, CF3COO-
(CsI)nI-
(Y(HCOO)3)nHCOO-

Figure 18

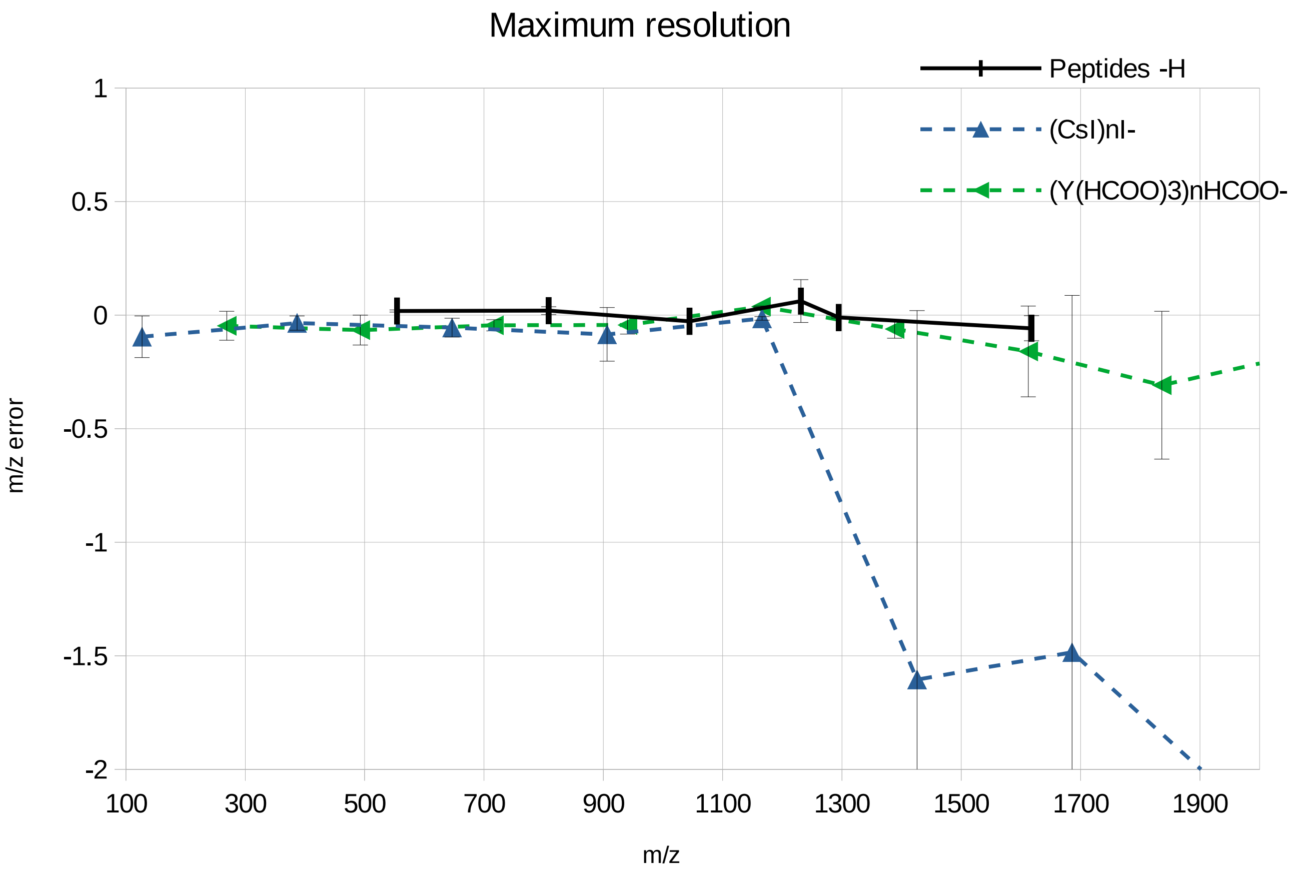

Figure 19

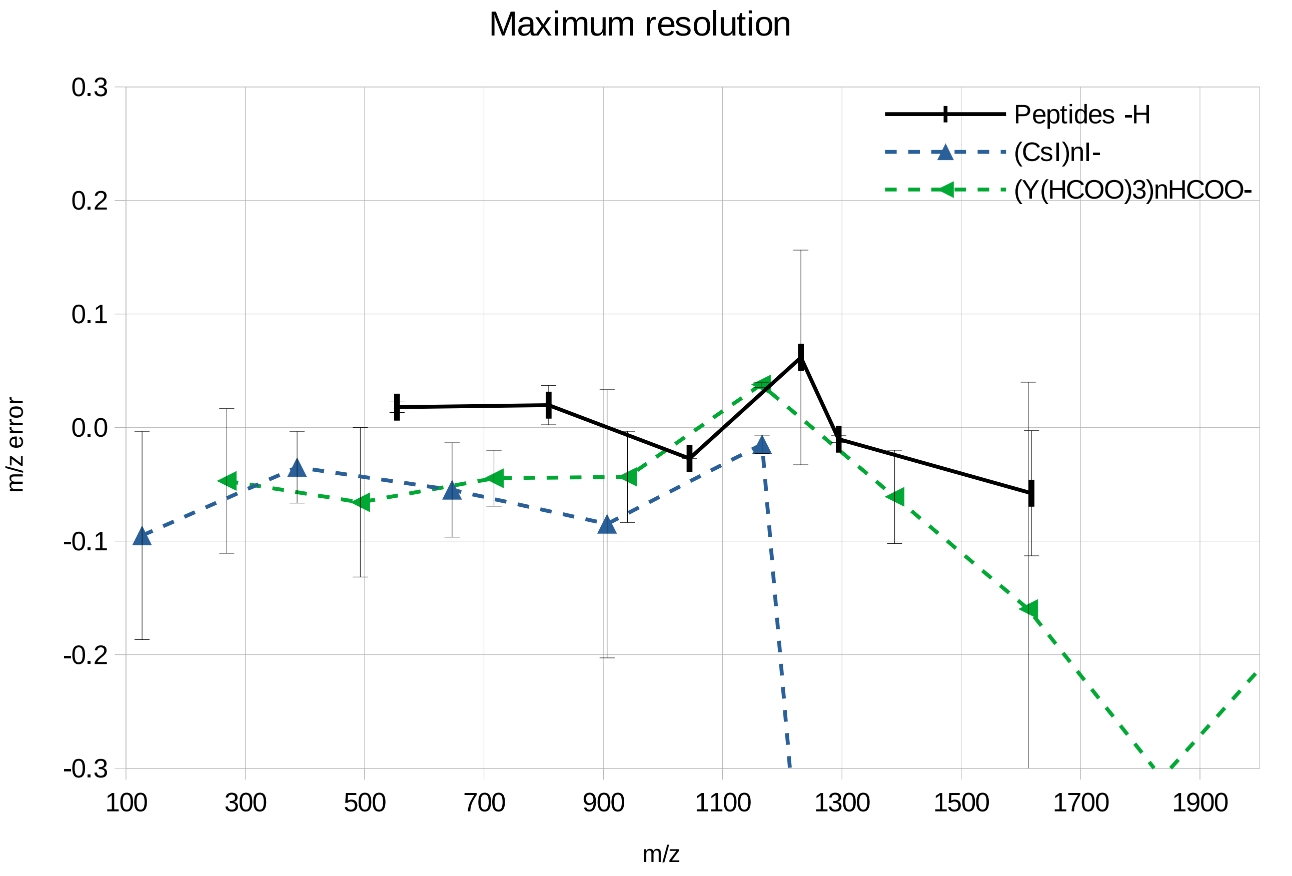

Figure 20
