# Supplementary material for: Chemical mass shifts of cluster ions and adduct ions in quadrupolar ion traps revisited and extended
Source: Rapid Commun Mass Spectrom. 2022 Dec 14;37(3):e9436. doi: 10.1002/rcm.9436 (PMC10078176; doi:10.1002/rcm.9436)
Supplement: Supplementary file 3 — DATA S3 Supplementary peak shapes [file RCM-37-0-s001.pdf]

Supplementary - peak shapes

Peak shapes not presented in the paper.

Please disregard *m/z* values as peak shapes are here shown *before* recalibration (the average *m/z* values after recalibration are in the charts in the paper and Supplementary mass shifts). Also disregard any charge state indications (unless otherwise stated, signals are singly charged). All spectra shown are the averages of several individual spectra. No smoothing has been applied.

Table 1: List of figures of peak shapes

Experiment	Analytes	Charge carrier	Fig. - peak shapes	
			LXQ LIT	amaZon QIT
Low mass range	Y(HCOO)3	+ $HCOO^-$		Sup. Fig. 2, 12, 22, 33
	NaCF3COO	+ Na^+		Sup. Fig. 1, 11, 21, 32
	ESI Tuning mix	+ H^+		Fig. 3, column 1<br>Sup. Fig. 6, 16, 26, 27, 37, 38
		+ CF_3COO^-		Sup. Fig. 3, 1, 23, 34
	Pierce calibration mixture	+ H^+	Fig. 4, column 1	
	CsI	+ Cs^+	Fig. 4, column 1	Fig. 3, column 6<br>Sup. Fig. 4, 5, 14, 15, 24, 25, 35, 36
	High mass dendrimer mixture	+ Na^+		Fig. 3, Column 2, 4<br>Sup. Fig. 7, 9, 19, 17, 28, 30, 39, 41
		+ Cs^+		Fig. 3, Column 3, 5<br>Sup. Fig. 8, 10, 18, 20, 29, 31, 40, 42
High mass range	High mass dendrimer mixture	+ Na^+	Fig. 5, row 1, 3, 5, 7<br>Sup. Fig. 54,	Sup. Fig. 43, 45, 47, 49
		+ Cs^+	Fig. 5, row 2, 4, 6 ,8<br>Sup. Fig. 54,	Sup. Fig. 44, 46, 48, 50
	CsI	+ Cs^+<br>+ 2 Cs^+		Sup. Fig. 51, 52

1.1 Xtreme-scan

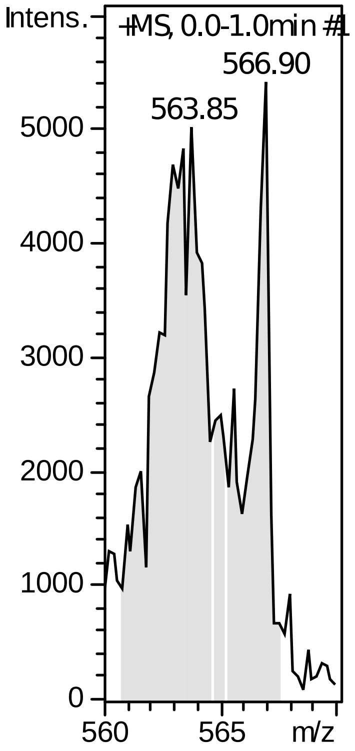

Figure 1 $NaCF_3COO$-cluster, positive mode, 10 *m/z* shown.

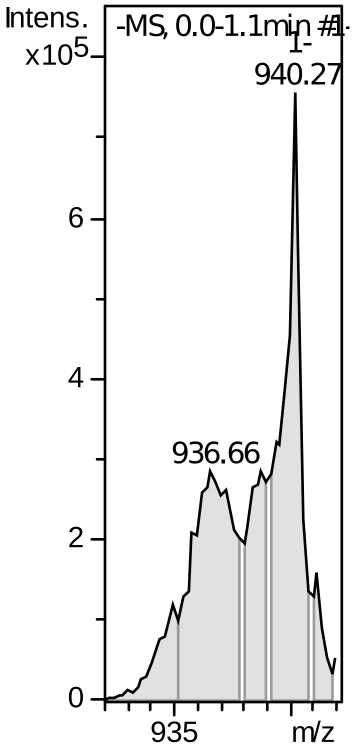

Figure 2 $Y(HCOO)_3^-$ cluster, negative mode, 10 *m/z* shown.

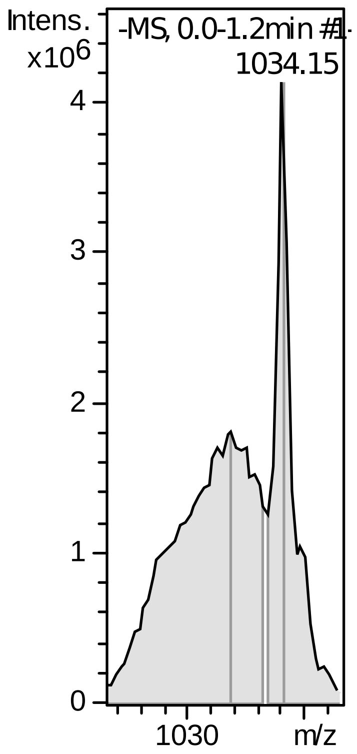

Figure 3 Tuning mix CF_3COO^--adduct, 10 *m/z* shown.

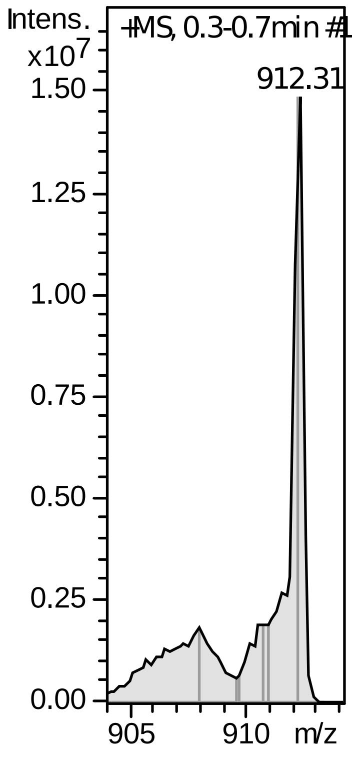

Figure 4 CsI-cluster, positive mode, 10 *m/z* shown.

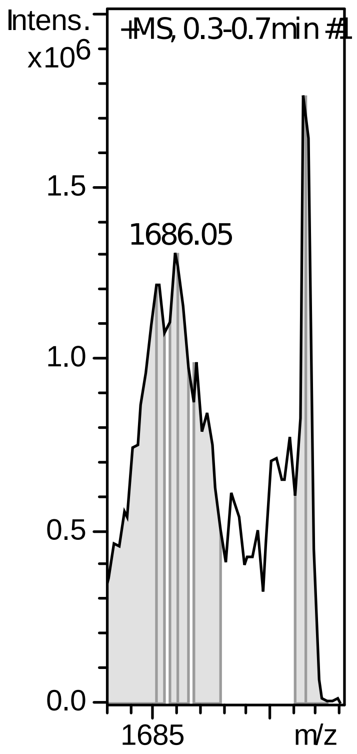

Figure 5 CsI-cluster, positive mode, 10 *m/z* shown.

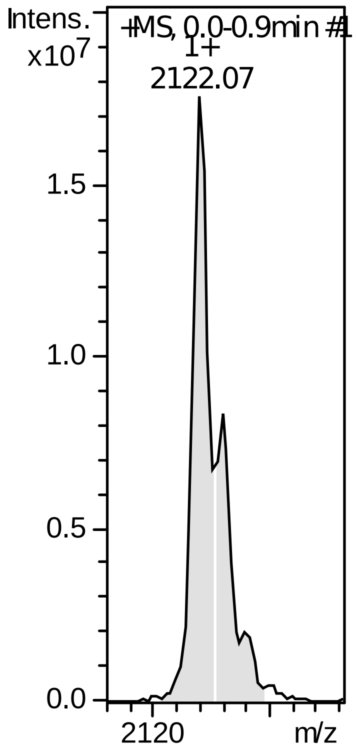

Figure 6 Tuning mix, positive mode, 10 *m/z* shown.

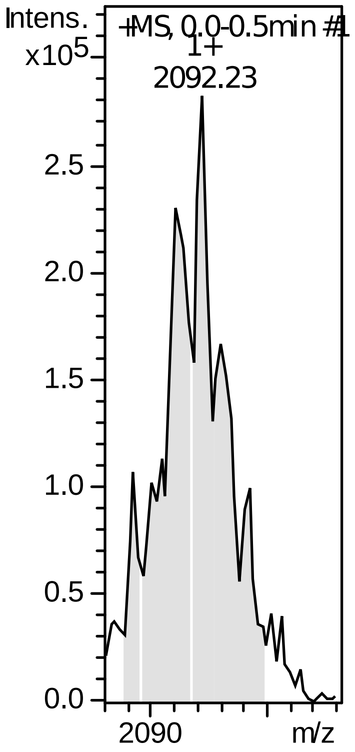

Figure 7 D2089 Na^{+}-adduct, 10 *m/z* shown.

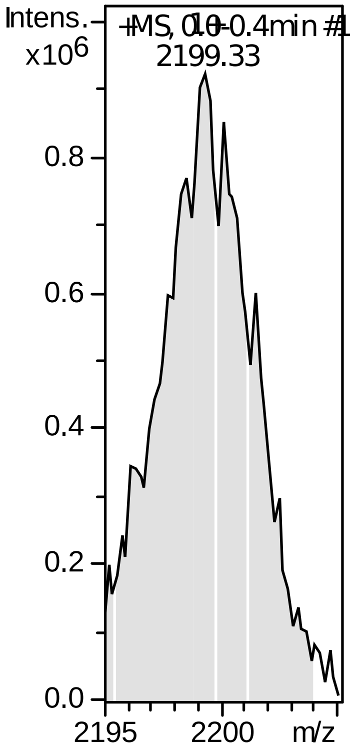

Figure 8 D2089 Cs^{+}-adduct, 10 *m/z* shown.

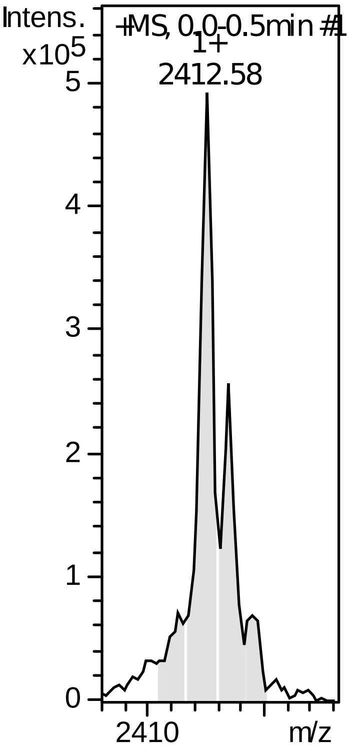

Figure 9 D2412 Na^{+}-adduct, 10 *m/z* shown.

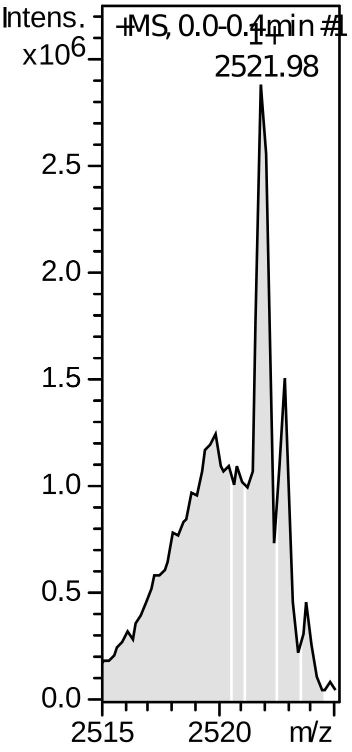

Figure 10 D2412 Cs^{+}-adduct, 10 *m/z* shown.

1.2 Ultra-scan

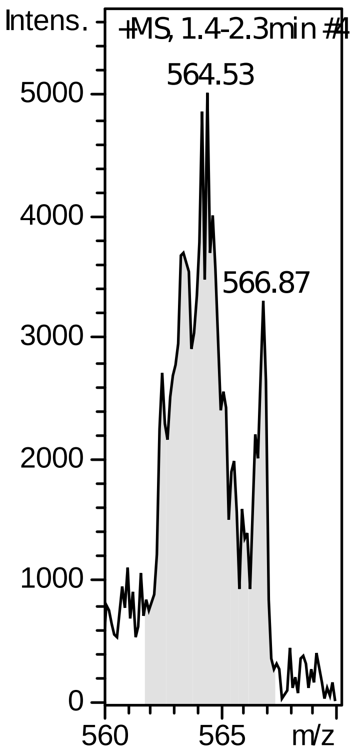

Figure 11 $NaCF_3COO$-cluster, positive mode, 10 *m/z* shown

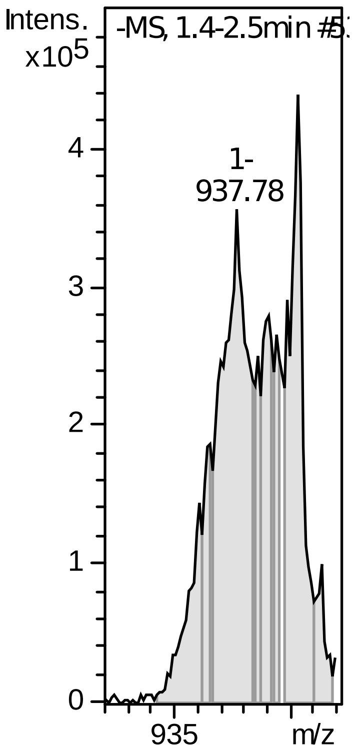

Figure 12 $Y(HCOO)_3$-cluster, negative mode, 10 *m/z* shown.

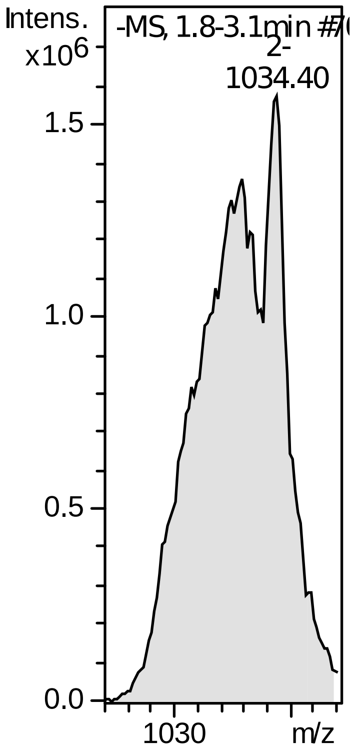

Figure 13 Tuning mix CF_3COO^--adduct, 10 *m/z* shown.

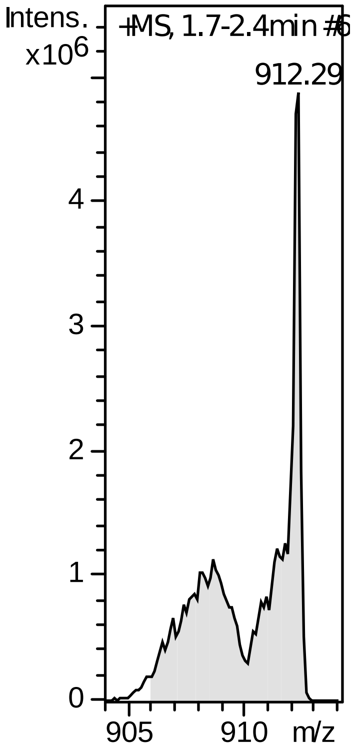

Figure 14 CsI-cluster, positive mode, 10 *m/z* shown.

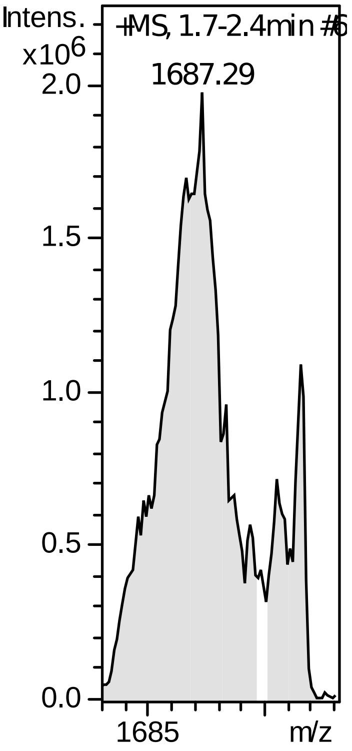

Figure 15 CsI-cluster, positive mode, 10 *m/z* shown.

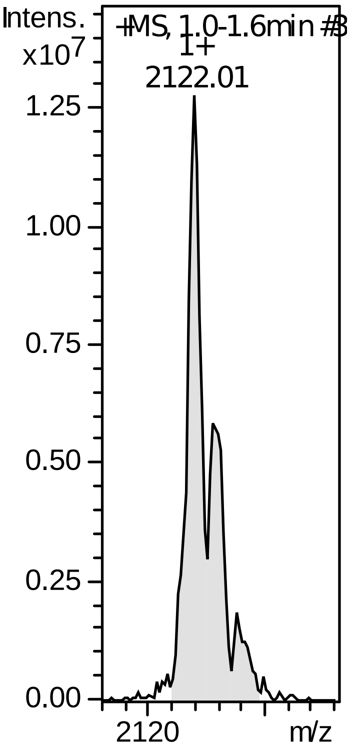

Figure 16 Tuning mix, positive mode, 10 *m/z* shown.

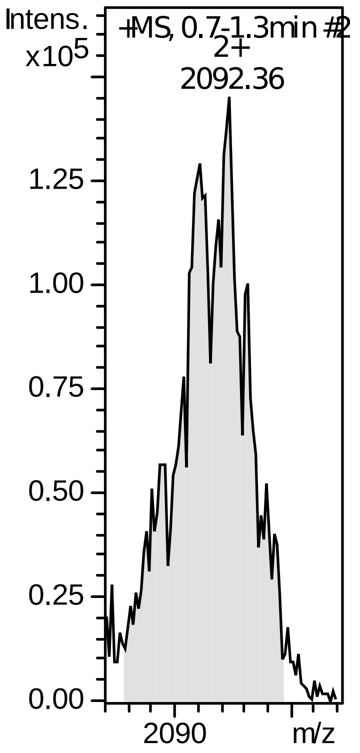

Figure 17 D2089 Na^{+}-adduct, 10 *m/z* shown.

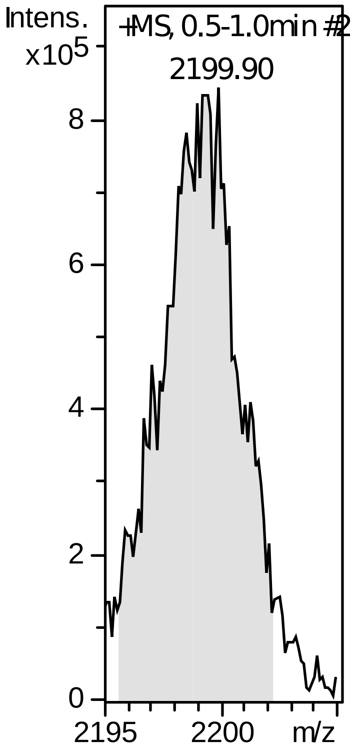

Figure 18 D2089 Cs^{+}-adduct, 10 *m/z* shown.

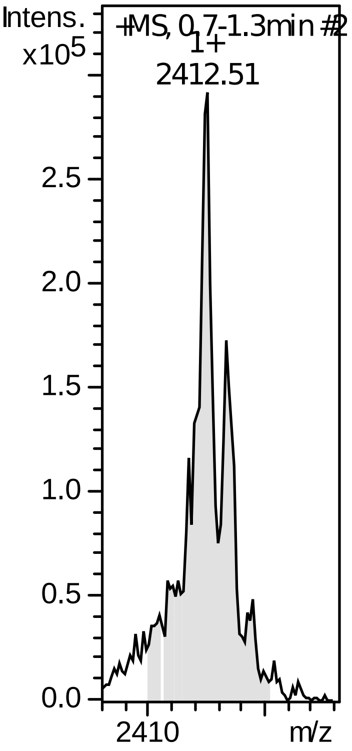

Figure 19 D2412 Na^+-adduct, 10 *m/z* shown.

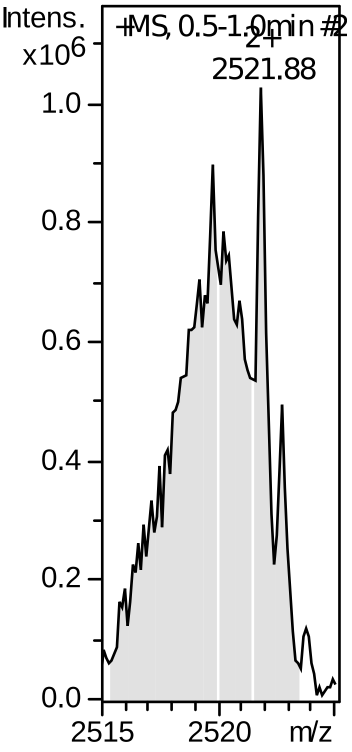

Figure 20 D2412 Cs^+-adduct, 10 *m/z* shown.

1.3 Enhanced resolution-scan

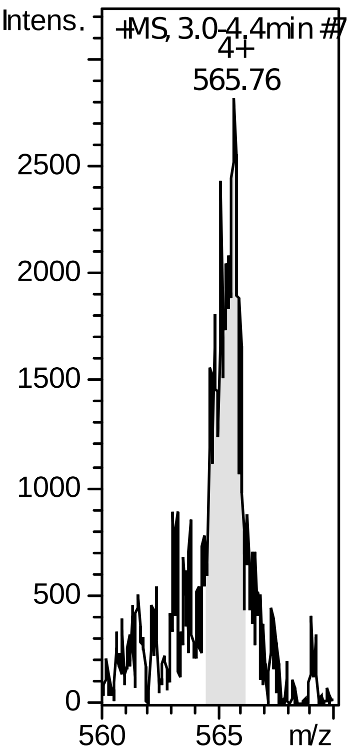

Figure 21 $NaCF_3COO$-cluster, positive mode, 10 *m/z* shown

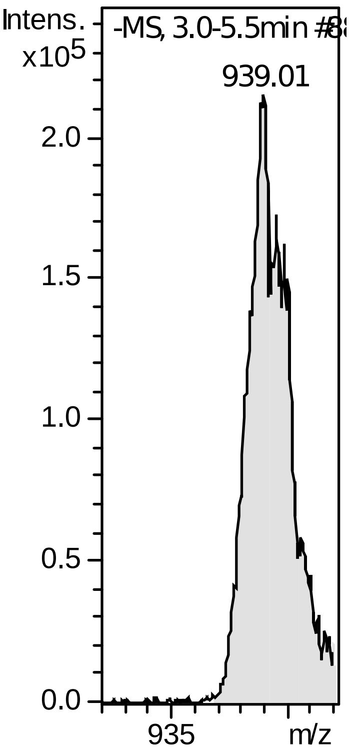

Figure 22 $Y(HCOO)_3$-cluster, negative mode, 10 *m/z* shown.

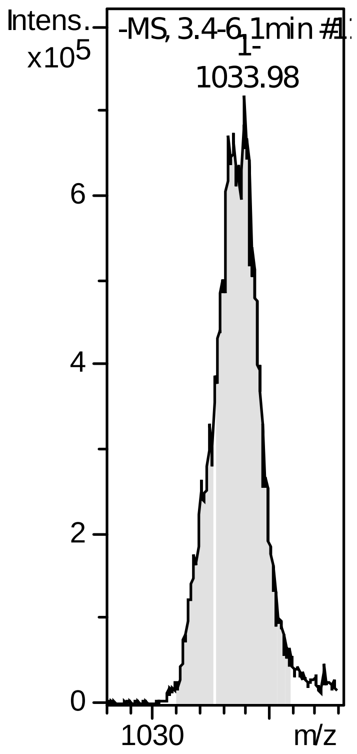

Figure 23 Tuning mix CF_3COO^--adduct, 10 *m/z* shown.

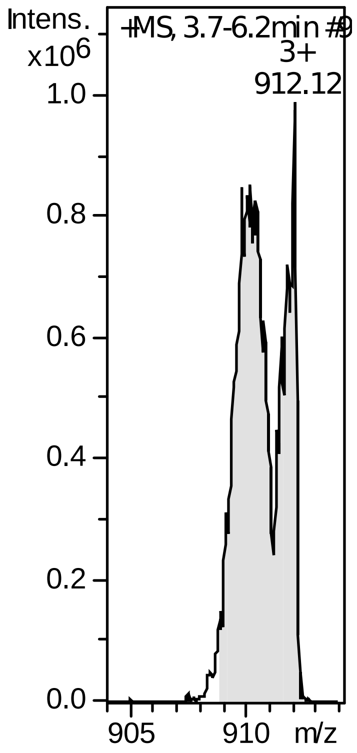

Figure 24 CsI-cluster, positive mode, 10 *m/z* shown.

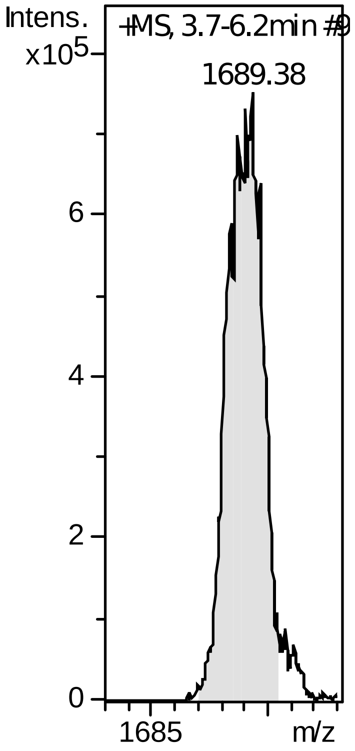

Figure 25 CsI-cluster, positive mode, 10 *m/z* shown.

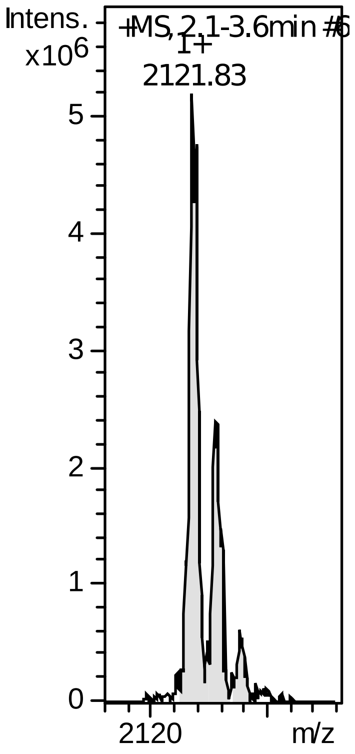

Figure 26 Tuning mix, positive mode, 10 *m/z* shown.

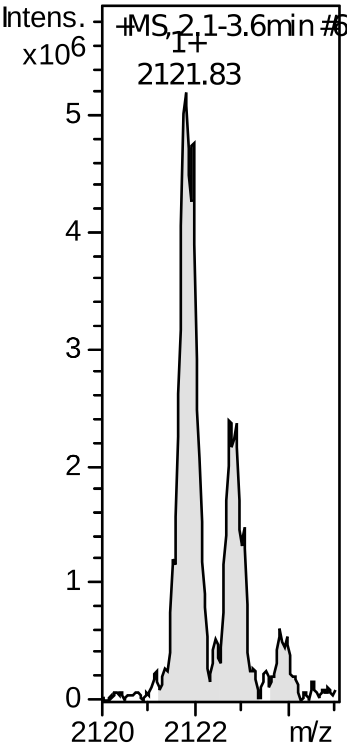

Figure 27 Tuning mix, positive mode, 5 *m/z* shown.

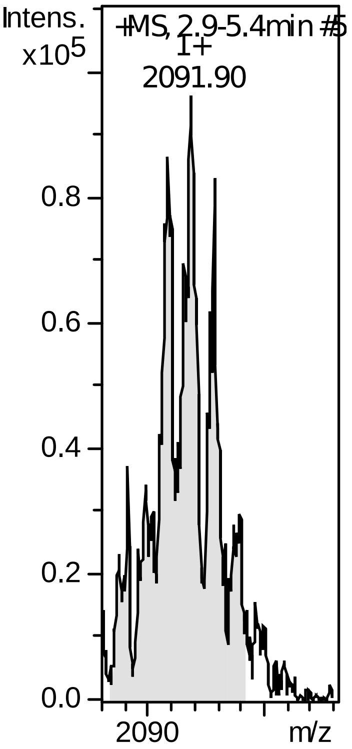

Figure 28 D2089 Na^+-adduct, 10 *m/z* shown.

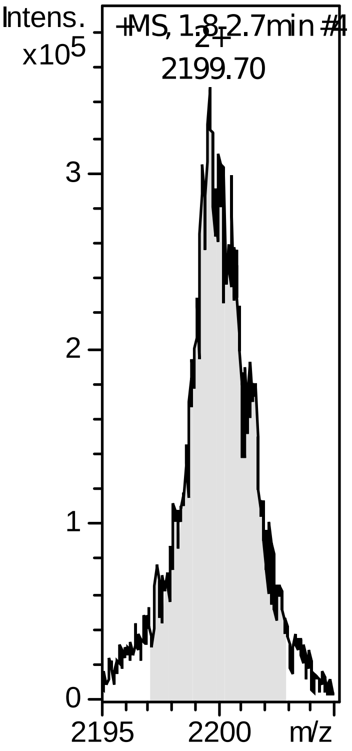

Figure 29 D2089 Cs^{+}-adduct, 10 *m/z* shown.

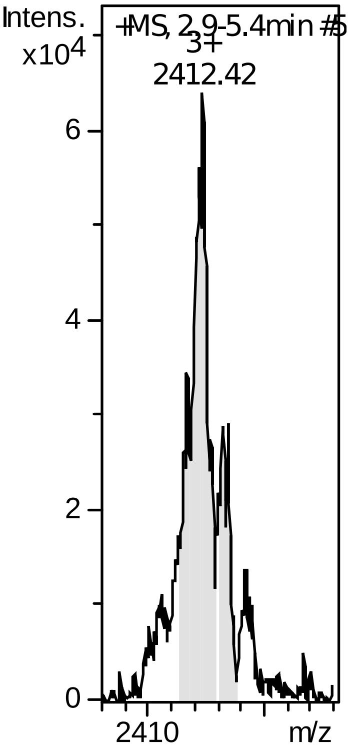

Figure 30 D2412 Na^{+}-adduct, 10 *m/z* shown.

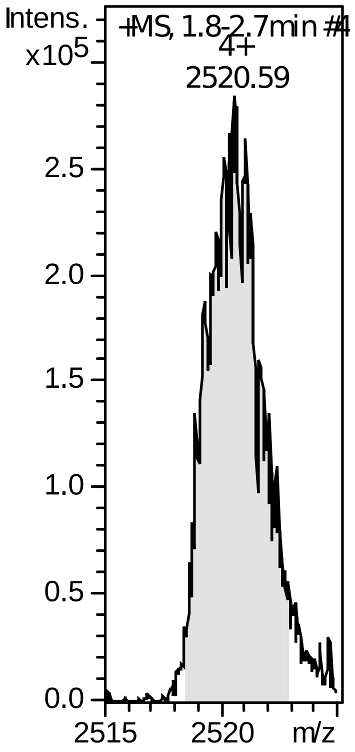

Figure 31 D2412 Cs^{+}-adduct, 10 *m/z* shown.

1.4 Maximum resolution-scan

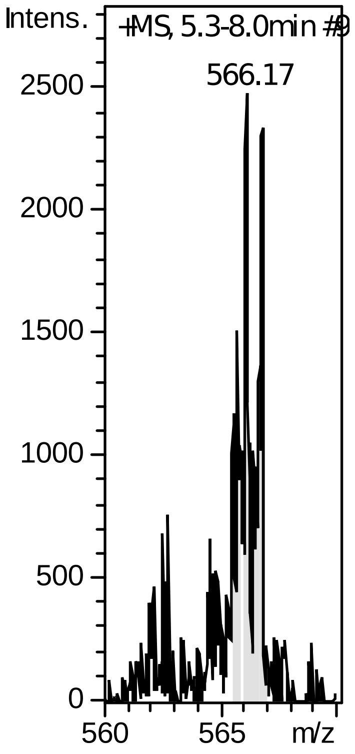

Figure 32 $NaCF_3COO$-cluster, positive mode, 10 *m/z* shown

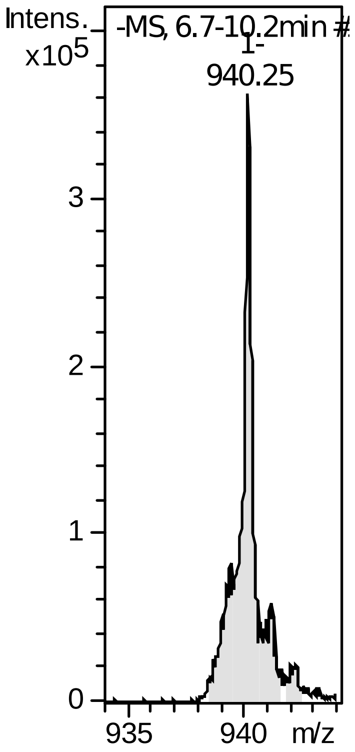

Figure 33 Y(HCOO)$_3$-cluster, negative mode, 10 *m/z* shown.

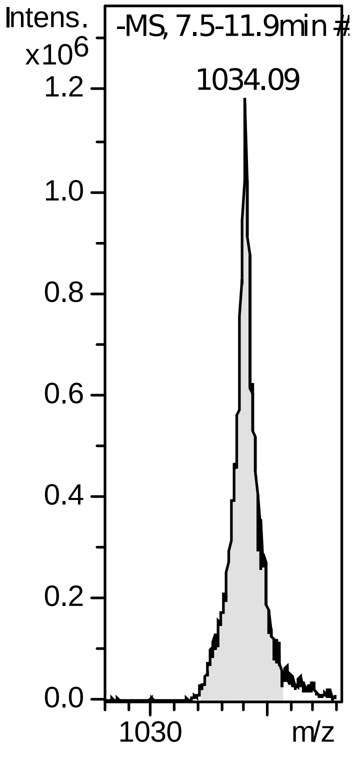

Figure 34 Tuning mix CF_3COO^--adduct, 10 *m/z* shown.

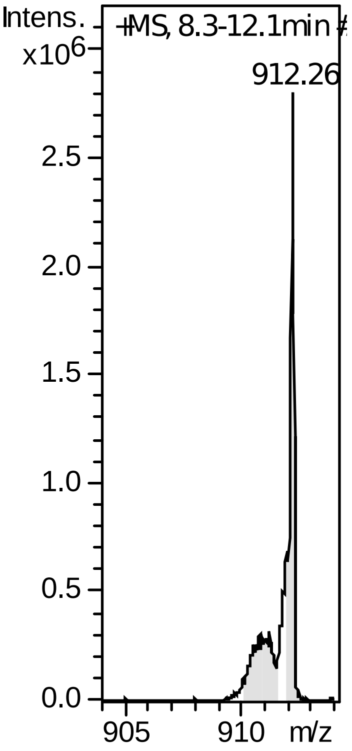

Figure 35 CsI-cluster, positive mode, 10 *m/z* shown.

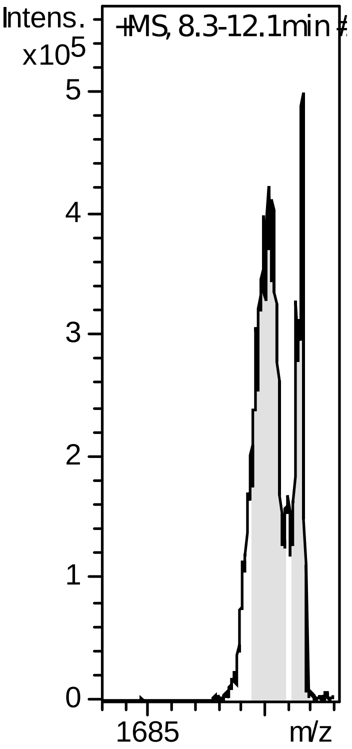

Figure 36 CsI-cluster, positive mode, 10 *m/z* shown.

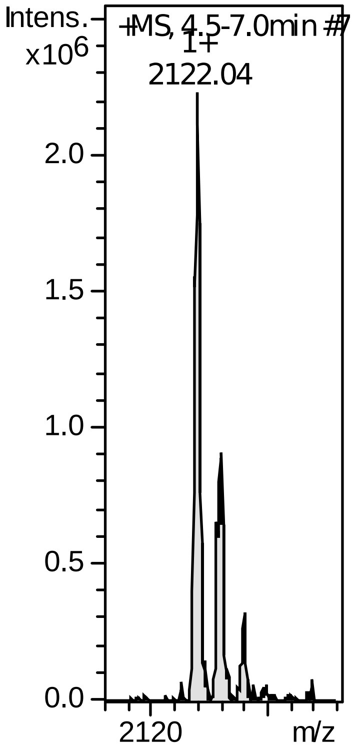

Figure 37 Tuning mix, positive mode, 10 *m/z* shown.

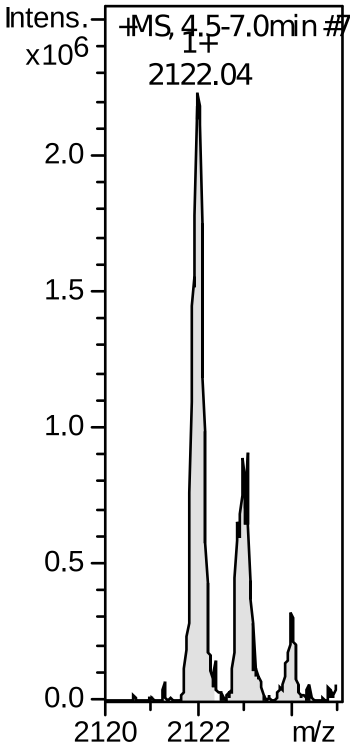

Figure 38 Tuning mix, positive mode, 5 *m/z* shown.

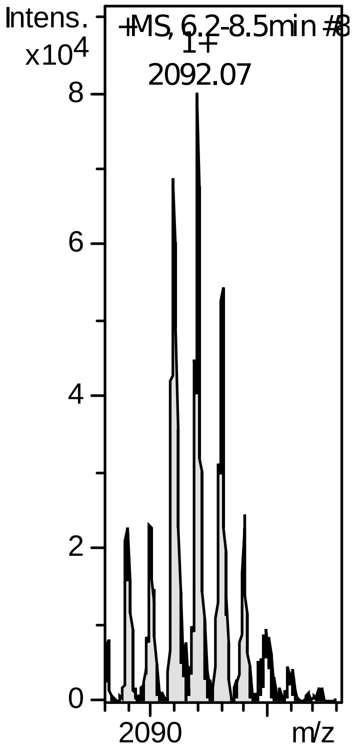

Figure 39 D2089 Na^{+}-adduct, 10 *m/z* shown.

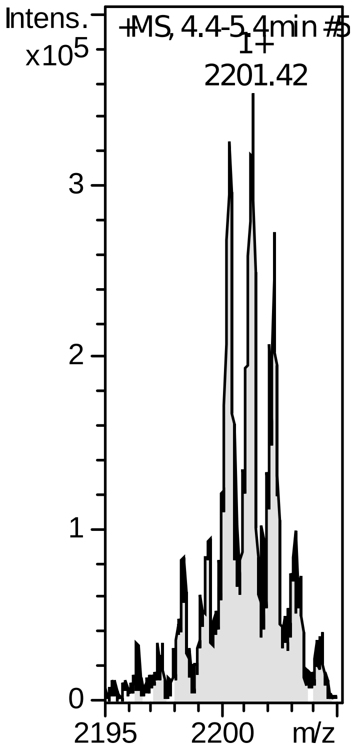

Figure 40 D2089 Cs^{+}-adduct, 10 *m/z* shown.

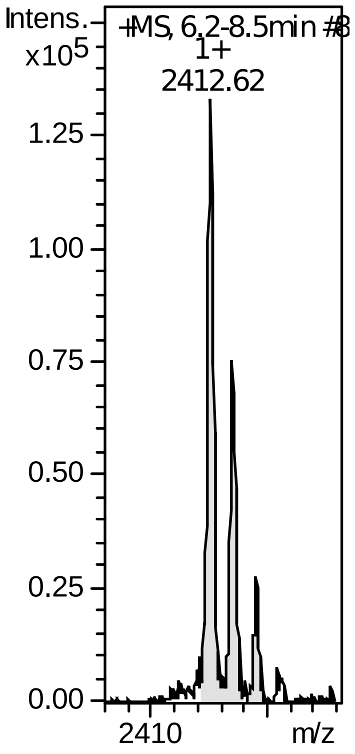

Figure 41 D2412 Na^{+}-adduct, 10 *m/z* shown.

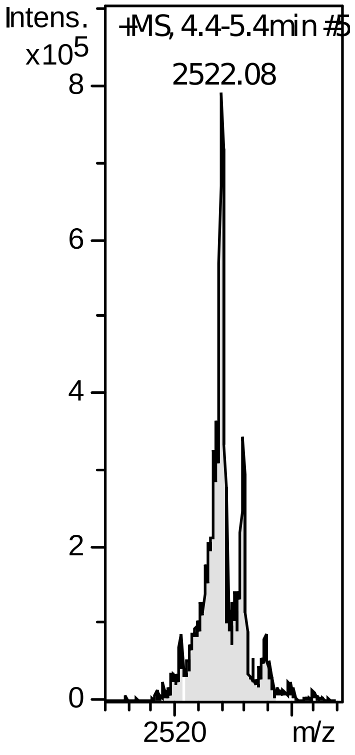

Figure 42 D2412 Cs^{+}-adduct, 10 *m/z* shown.

1.5 Extended mass range scan

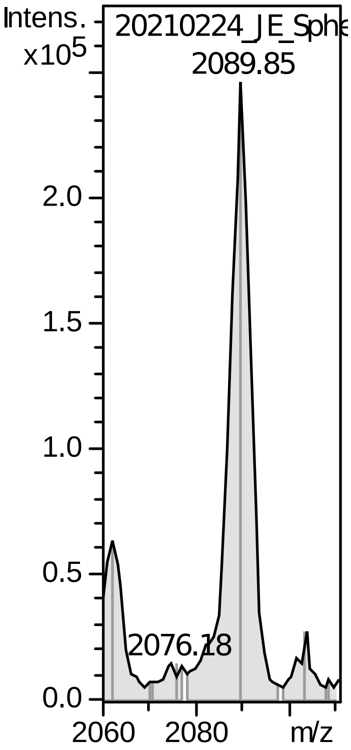

Figure 43 [D2089 + Na^{+}] 50 m/z shown.

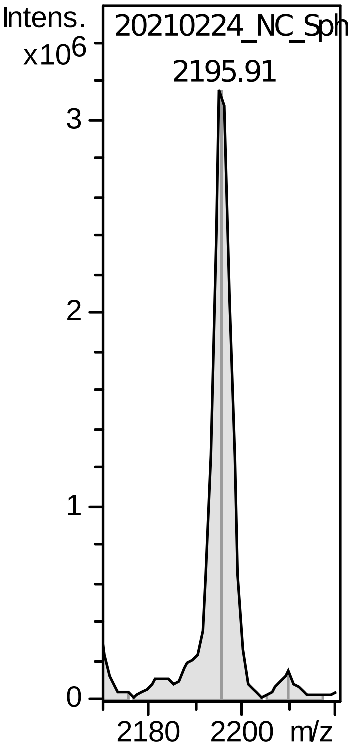

Figure 44 [2089 + Cs^{+}] 50 m/z shown.

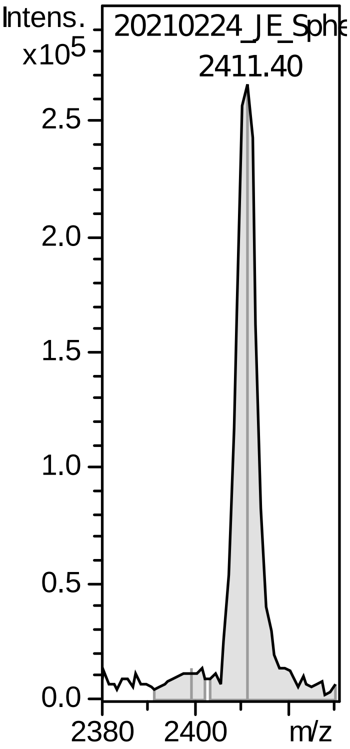

Figure 45 [2412 + Na^+] 50 m/z shown.

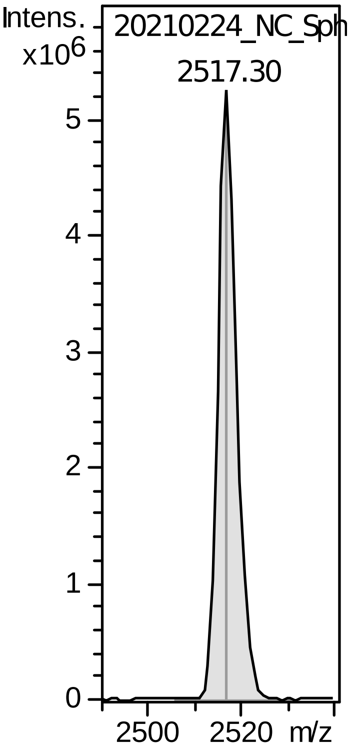

Figure 46 [2412 + Cs^+] 50 m/z shown.

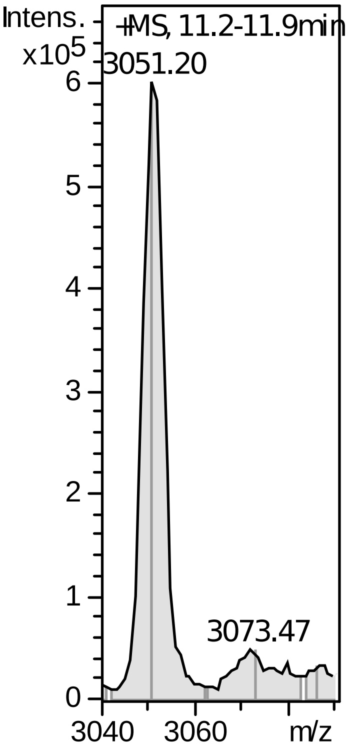

Figure 47 [D3055 + Na⁺] 50 m/z shown.

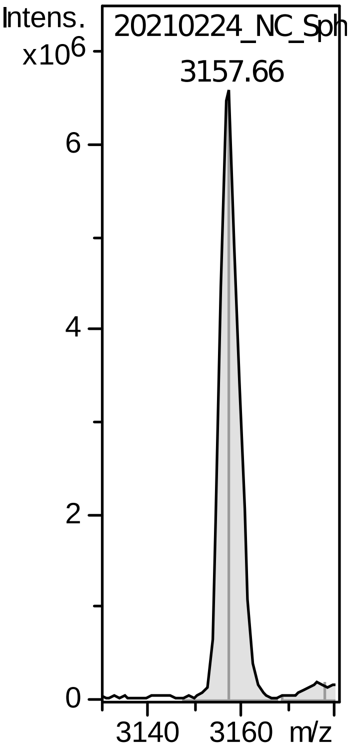

Figure 48 [D3055 + Cs⁺], 50 m/z shown.

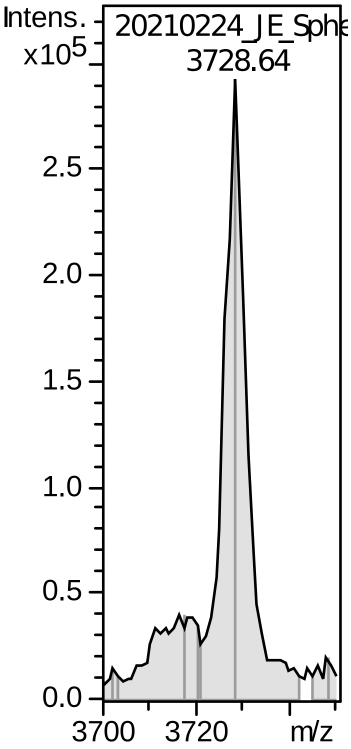

Figure 49 [D3732 + Na⁺] 50 m/z shown.

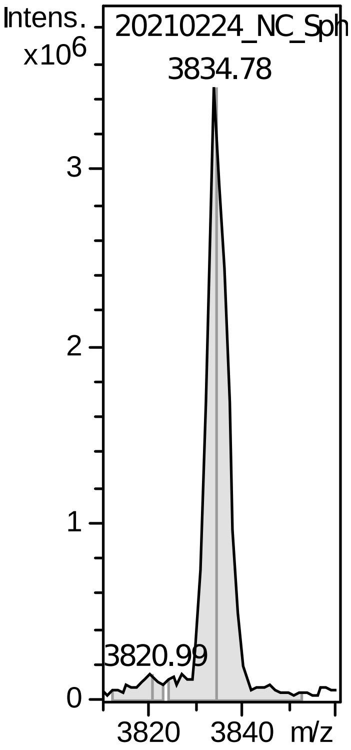

Figure 50 [D3732 + Cs⁺], 50 m/z shown.

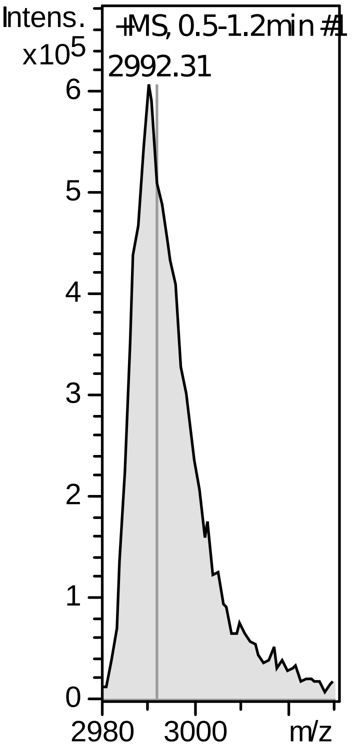

Figure 51 CsI+ ,50 m/z shown.

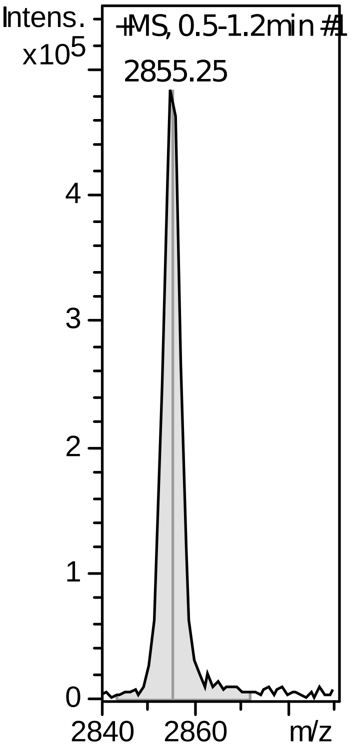

Figure 52 CsI 2+ , 50 m/z shown.

2 Peak shapes – Finnigan LXQ LIT high mass range

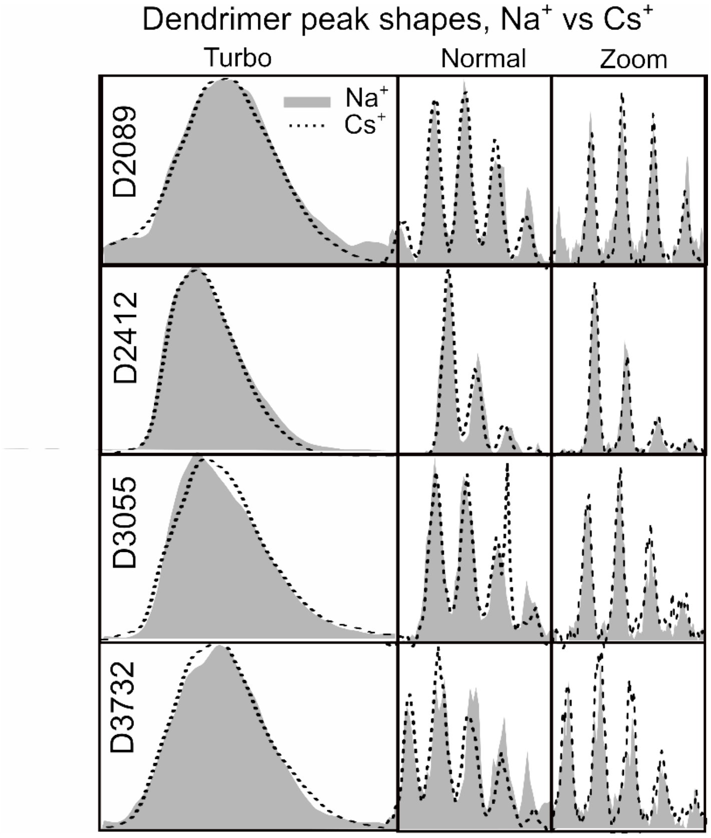

Figure 53. Peak shape comparison, Na^{+}- and Cs^{+}-adducted dendrimers. The *m/z* scale is the same for all spectra, but peaks are shifted in relative position and intensity for illustrative purposes.
